# A scalable molten-salt epitaxy of analog-compatible correlated perovskites at micrometer-scale thickness

**DOI:** 10.1093/nsr/nwag216

**Published:** 2026-04-08

**Authors:** Yi Bian, Peiheng Jiang, Nuofu Chen, Hao Zhang, Binghui Ge, Hongliang Dong, Jiaou Wang, Ho-Kwang Mao, Lidong Chen, Jikun Chen

**Affiliations:** Beijing Advanced Innovation Center for Materials Genome Engineering, School of Materials Science and Engineering, University of Science and Technology Beijing, Beijing 100083, China; School of Physics, MOE Key Laboratory for Nonequilibrium Synthesis and Modulation of Condensed Matter, Xi’an Jiaotong University, Xi’an 710049, China; School of New Energy, North China Electric Power University, Beijing 102206, China; Beijing Advanced Innovation Center for Materials Genome Engineering, School of Materials Science and Engineering, University of Science and Technology Beijing, Beijing 100083, China; State Key Laboratory of Opto-Electronic Information Acquisition and Protection Technology, Institute of Physical Science and Information Technology, Anhui University, Hefei 230601, China; Center for High Pressure Science and Technology Advanced Research, Shanghai 201203, China; Beijing Synchrotron Radiation Facility, Institute of High Energy Physics, Chinese Academy of Sciences, Beijing 100049, China; Center for High Pressure Science and Technology Advanced Research, Shanghai 201203, China; Shanghai Institute of Ceramics, Chinese Academy of Sciences, Shanghai 200050, China; Beijing Advanced Innovation Center for Materials Genome Engineering, School of Materials Science and Engineering, University of Science and Technology Beijing, Beijing 100083, China

**Keywords:** correlated oxides, perovskites, metastable materials, metal–insulator transition

## Abstract

Correlated perovskites display extraordinary analog functionalities catering to neuromorphic computing and advanced field perceptions, transcending post-Moore limitations. Nevertheless, it is yet infeasible to achieve scalable material growth of correlated perovskites at micrometer-scale thicknesses, a prerequisite for enabling device resistances compatible with analog circuit requirements. Herein, we demonstrate ultra-effective growth of archetypal metastable correlated perovskites, e.g. nickelates (*RE*NiO_3_), via liquid-phase epitaxy within alkali-chloride molten salts, realizing micrometer-scale thickness and scalability. The molten salts provide an ultra-stable thermodynamic environment and consistent ionic-precursor availability for long-period oriented growth, effectively enabling stacking faults formation to mitigate high-magnitude lattice mismatches. This bridges current technological gaps in micrometer-thick film growth of *RE*NiO_3_ and achieves record-competitive electronic phase transitions at analog-compatible resistances, enabling more effective and energy-efficient analog cryogenic alarming applications. For the first time, wafer-scale *RE*NiO_3_ with high uniformity was successfully grown within low-melting-point eutectic alkali chlorides, eliminating previous reliance on MPa-high oxygen pressures. Our strategy was extendable to multiple oxide systems, covering diverse functionalities, e.g. colossal magnetoresistance, oxide electrodes, and superconductivity.

## Teaser

Wafer-scale growths of metastable perovskite nickelates at micrometer-scale thickness were achieved via alkali-chloride molten salts assisted liquid phase epitaxy. This bridges current technological discontinuities in film depositions, enabling analog-compatible and scalable correlated electronic applications.

## INTRODUCTION

The intricate *d*-orbital electronic phase diagram in correlated perovskite oxides gives rise to rich quantum phenomena, primarily driven by strong electron correlation effects, providing an analog paradigm to transcend performance limitations of post-Moore semiconductor devices [1–3]. This is evidenced by the exceptional performance of correlated perovskite nickelates (*RE*NiO_3_) in neuromorphic computing and advanced field perceptions, grounded in multiple Ni-3*d* related electronic phase transitions via field manipulations of their electron Coulomb energy (*U*) [4,5]. Among existing correlated systems, the *RE*NiO_3_ exhibits metal–insulator transitions (MITs) with the highest tunability, stemming from their high tolerance for distorting the NiO_6_ octahedra for broad regulatory scope of Ni-3*d*/O-2*p* orbital overlapping and its potential to (un)shield *U* [6,7]. Consequently, an extraordinarily wide range of critical temperatures (*T*_MIT_) within 90–600 K is enabled for *RE*NiO_3_ via simple rare-earth substitutions [8,9], enabling promising applications, such as critical temperature resistor (CTR) [10], correlated logical devices [2], passive laser protection [11], and infrared camouflage [4]. Beyond conventional Mott-type MIT via switching between manifestation and shielding of *U*, the strength of *U* within *RE*NiO_3_ is more directly adjustable from the perspective of Ni 3*d* orbital occupations via Mottronic protonation [7,12]. These result in broadening of the band gap (*E*_g_) via further formation of hundreds of sub-states between electron itinerant Ni^3+^($t_{2g}^6e_g^1$) and electron localized Ni^2+^($t_{2g}^6e_g^2$), consuming merely femtojoules of energy [2,13]. Harnessing the resultant extensive resistive modulations to exploit analog neuromorphic simulations for neural networks [2,14] or bionic sensing systems [7,12] has realized a quantum leap in computability and energy efficiency, rivaling biological brains [2,13,14].

Nevertheless, the analog electronic applications of correlated perovskites are constrained by their currently ineffective material growths, in terms of fidelity and scalability [5,15]. From the one side, limitation in fidelity stems from the absence of a feasible avenue to fabricate correlated perovskites at micrometer-scale thickness [16], a critical scale to enable their device resistances as compatible with efficient analog circuit requirements (e.g. 10^0^–10^4^ Ω/□) [17], in view of their resistivities (e.g. 10^−6^ to 10^−2^ Ω m) [10,18,19]. Yet, the micrometer-thick material fabrications situate at the juncture of technological discontinuity, as it is too thick for ‘bottom-up’ film growths [16] and too thin for ‘top-down’ processing from the bulk [20]. Presently, the achievable device resistance for *RE*NiO_3_ is either too high (e.g. >10^4^ Ω) [16] for 10^2^-nm-thick thin films or too low for bulk pellets (e.g. <10^−1^ Ω) [21], impeding an efficient analog electronic application in integrated circuits (IC) or discrete electronic devices. From the other side, the scalability in high-quality film growths of correlated perovskites is struggled to source a lattice matching substrate at wafer scales [5]. Also, the inherent metastability of some highly promising perovskite systems (e.g. *RE*NiO_3_) also challenges their scalability in growth [5,9,22], as it requires MPa-high oxygen pressures (*p*_O_2__) to counteract their positive formation free energies (Δ*G*) [14,22]. Unlike the present scalable film growths in vacuum (e.g. chemical vapor deposition, metal organic chemical vapor deposition) or under ambient pressure (e.g. conventional spin coating), the MPa gaseous pressures at high temperatures pose critical complications and safety hazard for amplified growths of *RE*NiO_3_. To date, wafer-scale growth remains unattainable for the metastable *RE*NiO_3_, thereby depriving these materials of a critical competitive advantage over their post-Moore electronic roadmap counterparts with scalability in growth, such as MoS_2_ and WS_2_ [23,24]. To bridge the gap between the fundamental research frontier and practical electronic applications of correlated perovskites, it is urgent to develop an effective avenue that enables their scalable growth at micrometer-scale thickness.

In this work, we demonstrate an alkali-chloride-assisted liquid-phase epitaxy (LPE) that achieves micrometer-thick growths of archetypal correlated perovskites with ultra-high effectiveness and scalability, even for metastable ones such as *RE*NiO_3_. The alkali-chloride molten salts provide stable thermodynamic environment, steady ionic precursors, and low epitaxy temperatures to counteract inherent metastability and lattice mismatch in long-period oriented growths. Consequently, the preferential crystal orientation sustains at micrometer-scale thickness for as-grown *RE*NiO_3_, enabling MIT and Mottronic functionalities rivaling their best reports at an analog-compatible device resistance. By encapsulating micrometer-thick *RE*NiO_3_ over external oxide supporters, we developed a series of miniaturized CTR discrete devices, modules, and systems, realizing widely adjustable critical temperature (*T*_C_) from 120 to 300 K. This effectively fills the ‌technological void in CTRs at cryogenic temperature ranges, giving rise to a quantum leap in sensitivity and energy efficiency for cryogenic alarming. To further pave the way to their prospective IC analog applications, we achieved wafer-sized growth of *RE*NiO_3_ on sapphires via low-temperature LPE within eutectic molten salt, eliminating their previous reliance on MPa-high *p*_O_2__. The generality of our LPE strategy was further demonstrated by its extendibility to growth of other multicomponent oxides systems (e.g. Pr_0.67_Sr_0.33_MnO_3_, SrRuO_3_, and Bi_2_Sr_2_CaCu_2_O_8_), covering a large variety of functionalities.

## RESULTS AND DISCUSSIONS

### Liquid phase epitaxy of metastable perovskites within molten alkali chlorides

Realizing oriented growth of correlated perovskites (e.g. *RE*NiO_3_) over large thickness is conventionally obstructed by the inevitable thermodynamic fluctuation during long periods. This is exemplified in *RE*NiO_3_ that relies on post annealing of their pre-deposited cation precursors at MPa-high *p*_O_2__ for crystallization [7,14], impeding their scalable growth. Also, the post-annealing results in a distinct thermodynamic landscape adjacent to and far away from a coherent interface with the substrate. Consequently, the oriented growth is only sustained within ∼10^1^ nm thickness from the interface, while precursors located further away usually homogeneously crystallized, showing polycrystallinity and less abrupt MITs [7,25].

**Figure 1.**
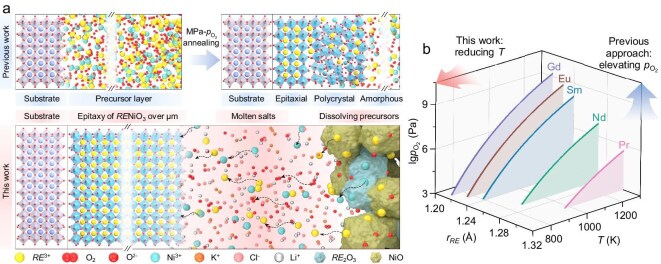
Molten alkali chlorides LPE of correlated perovskites. (a) Schematic illustration of an alkali-chloride molten-salt-assisted LPE enabling micrometer-thick growths of *RE*NiO_3_. The upper two figures show the two steps in previous strategies to grow metastable *RE*NiO_3_ films: (left) pre-depositing cation precursors and (right) post-annealing at MPa-high oxygen pressure for crystallization into perovskites. Consequently, the thermodynamic environment for precursors located adjacent to the substrate differs from the ones located far away, giving rise to a limitation in the thickness of oriented growth as shown by the upper right figure. In contrast, in our alkali-chloride molten salt-assisted low-temperature LPE, the supply of precursors concurrently collaborates with their crystallization within the molten salts. The molten alkali chloride gradually dissolves the *RE*_2_O_3_ and NiO, as limited by solubilities, to steadily supply ionic *RE*^3+^ and Ni^3+^ at constant concentrations at the surface region at low temperatures, thus creating a stable thermodynamic environment to sustain long-period oriented growth beyond the micrometer scale. (b) Thermodynamic equilibrium relationship between oxygen pressure (*p*_O2_) and reaction temperature (*T*) for *RE*NiO_3_ with *RE* (e.g. *RE* = Pr, Nd, Sm, Eu, and Gd) at various ionic radii (*r*_RE_). The lines represent the *p*_O2_–*T* relationship when the formation free energy (Δ*G*) equals zero. The thermodynamic conditions provided by the upper left region of the lines are toward the synthesis of *RE*NiO_3_, while the bottom right one is toward its decomposition.

To realize oriented growth of correlated perovskite over larger thickness, we develop an alkali-chloride molten salt assisted low-temperature LPE process, through which much more stable thermodynamic environment and constant ionic precursors are provided sustaining long period epitaxy. As illustrated in Fig. 1a, the molten alkali chloride gradually dissolves the *RE*_2_O_3_ and NiO, as limited by solubilities, to steadily supply ionic *RE*^3+^ and Ni^3+^ at constant concentrations at the surface region at low temperatures. Furthermore, it is capable of descending the temperature of LPE down to exceptionally low magnitude (e.g. down to 350°C for using eutectic KCl/LiCl at 2/3) without sacrificing the kinetic perspective [26]. As the equilibrium *p*_O_2__–*T* relationships at Δ*G* = 0 shown in Fig. 1b for *RE*NiO_3_ with various *RE* (see more details in Fig. S1 and Table S1), the applied *p*_O2_ and *T* must fall within the upper-left sides (e.g. Δ*G* < 0) to allow synthesis. Thus, descending temperatures provides an alternative route to reduce Δ*G*, apart from involving high-*p*_O2_, shedding light on their scalable growth at ambient pressure.

### Enabling micrometer-thick oriented growth and underneath microscopic mechanism

**Figure 2.**
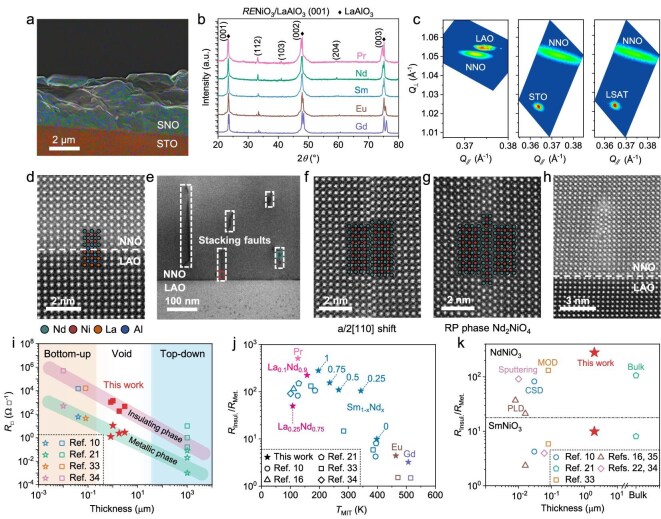
Enabling oriented growth of *RE*NiO_3_ at micrometer-scale thickness for analog-compatible MIT. (a) Representative cross-section morphology of as-grown *RE*NiO_3_ films via scanning electron microscopy. (b) The XRD patterns for *RE*NiO_3_/LaAlO_3_ (001) with various *RE*. (c) The RSMs of NdNiO_3_ grown on LaAlO_3_, SrTiO_3_, and (LaAlO_3_)_0.3_(Sr_2_AlTaO_6_)_0.7_ with (001) orientations. (d–h) HAADF morphologies of the NdNiO_3_/LaAlO_3_ (001). (d) Initially, the NdNiO_3_ displays a coherent interface with LaAlO_3_. (e) The interfacial coherency gradually relaxed with an increasing thickness of epitaxy via stacking faults, such as the displacement between adjacent rock-salt layers (right box) and inserting a rock-salt layer to form RP layered perovskites (left box). (f) The HAADF morphology of displacement between adjacent rock-salt layers. (g) The HAADF morphology of RP layered perovskites. (h) The interfacial morphology underneath the as-formed stacking faults. (i) Comparing the *R*_□_ of metallic and insulating *RE*NiO_3_ grown in this work to the previous ones achievable in their nanometer-thick thin films [10,33,34] and bulk pellets [21], hence bridging the present void in their effective μm-scale growth to enable analog-compatible *R*_□_. (j) Comparison of the magnitude in resistive switch (*R*_Insul._/*R*_Met._) across various *T*_MIT_ for *RE*NiO_3_ grown in this work to their previous reports [10,16,21,33,34]. (k) Comparing the *R*_Insul._/*R*_Met._ ever achievable for SmNiO_3_ and NdNiO_3_ grown in this work and previous reports [10,16,21,22,33–35] at various thicknesses.

Our LPE strategy was firstly applied to grow *RE*NiO_3_ on single crystalline perovskite substrates, such as LaAlO_3_ (LAO), SrTiO_3_ (STO), and (LaAlO_3_)_0.3_(Sr_2_AlTaO_6_)_0.7_ (LSAT) within KCl under MPa-high *p*_O2_. The as-grown films are a few micrometers thick, as indicated by their cross-section morphology shown in Fig. 2a (see more in Fig. S2). As their representative X-ray diffraction (XRD) patterns shown in Fig. 2b (see more results in Fig. S3), the film peaks appear mainly adjacent to the ones from the substrate. With a reducing ionic rare-earth radius (*r*_RE_), the film peaks shift rightward, owing to the reduction in the lattice constant of *RE*NiO_3_. Further reciprocal space mapping (RSM) shown in Fig. 2c indicates that such preferential oriented growths of *RE*NiO_3_ are sustained even at large lattice mismatches via gradual lattice relaxations across micrometer-thick epitaxy. This is more clearly indicated in Fig. S4, where the thickness dependence of the in-plane lattice relaxation is estimated from RSM. We further exclude the potential contaminations on as-grown films by the residual molten salts, by performing the X-ray photoelectron spectroscopy (XPS) analysis as results shown in Fig. S5.

To elucidate the underneath microscopic mechanisms for extending oriented growths of *RE*NiO_3_ beyond micrometer-thick, the high-angle annular dark field (HAADF) was performed to investigate their cross-section morphology, as shown in Fig. 2d–h (see more representative cases in Figs S6 and S7). The *RE*NiO_3_ adjacent to the interface was coherently grown on the substrate (Fig. 2d), while the stacking faults appear at several tens of nanometers away from the interface (Fig. 2e). Representatively, the displacements of *a*/2[110] between adjacent rock-salt layers are observed in Fig. 2f, while the insertion of rock-salt layers (e.g. *RE*O) forming a local region of Ruddlesden–Popper (RP) layered perovskite is demonstrated in Fig. 2g. This understanding is further confirmed by Fig. 2h, where a coherent interface underneath the stacking fault is observed. The stepwise formation of stacking faults mildly relaxes the lattice constants of *RE*NiO_3_ initially compliant to the substrate toward their bulk magnitudes [27,28], avoiding the previously sudden changes in thermodynamic conditions owing to more abruptly relaxed interfacial coherency. Hence, the preferential crystal orientation of *RE*NiO_3_ is sustainable over micrometer-scale thickness and across large magnitudes of lattice mismatches. The high crystallinity in as-grown *RE*NiO_3_ is further confirmed by a higher magnitude in the critical electric field to trigger its MIT compared to the previous report [29], as demonstrated in Fig. S8.

### Realizing record-competitive MIT functionality at analog-compatible resistances

Our LPE strategy further enables high tunability of the *RE* constituent within *RE*NiO_3_, and thereby their electronic structures. Figure S9 shows the near edge X-ray absorption fine structure (NEXAFS) spectroscopy of the Ni-*L*_3_ and O-*K* edges for as-grown *RE*NiO_3_ with various *RE* compositions. The Ni-*L*_3_ edge originates from the Ni-2*p*→Ni-3*d* transition and splits into two peaks, while the magnitude of their splitting reflects the charge-transfer gap [30,31]. As more clearly demonstrated in Fig. S9b, a more pronounced splitting of the Ni-*L*_3_ edge is observed for *RE*NiO_3_ with heavier *RE*, indicating their larger charge transfer gap and more strengthened insulating orbital configurations at more distorted NiO_6_ octahedra [30,31]. Further consistency is observed in the NEXAFS spectra for the O-*K* edge (see Fig. S9d), where the *RE*NiO_3_ with heavier *RE* shows smaller full width at half-maximum (FWHM) in its pre-peak owing to reduced orbital overlapping between Ni-3*d* and O-2*p* [30,32].

Consequently, widely adjustable MIT properties were achieved for as-grown *RE*NiO_3_ via substituting *RE* with La, Pr, Nd, Sm, Eu, and Gd, as demonstrated by their temperature dependent sheet resistance (*R*_□_–*T*) shown in Fig. S10. Their micrometer-scale thicknesses enable the optimum range of *R*_□_ for both insulating and metallic phases catering for direct analog electronic applications (e.g. 10^0^–10^4^ Ω/□), as previously unachievable, as compared in Fig. 2i [10,21,33,34]. It is also worth noticing that the resistivity of the presently grown *RE*NiO_3_ distributed within the range of the previously reported magnitudes, as demonstrated by Fig. S11. Their *T*_MIT_ and resultant resistive switch (*R*_Insul._/*R*_Met._) were further derived, as more details shown in Figs S10 and S12. A monotonic elevation in *T*_MIT_ from 100 to 500 K is observed with a reducing *r*_RE_ (see the inset of Fig. S10a), as is in consistency to the previous reports [10,16,21,33,34]. At similar *T*_MIT_, larger *R*_Insul._/*R*_Met._ were achieved for the presently grown *RE*NiO_3_ compared to the previous reports [10,16,21,33,34], as demonstrated in Fig. 2j. In particular, as-grown micrometer-thick SmNiO_3_ and NdNiO_3_ exhibit more abrupt MITs, compared to their previously reported counterparts including both the nanometer-thick films [10,16,22,33–35] and bulk pellets [21], as shown in Fig. 2k.

Figures S13–S15 further demonstrate the MIT properties for as-grown *RE*NiO_3_ films on more types of perovskite substrates at more orientations, e.g. LAO (110), LAO (111), STO (001), and LSAT (001), where similar MIT properties are observed compared to the ones grown on LAO (001). Apart from conventional MIT, the Mottronic functionalities triggered by protonation were also achieved in these micrometer-thick *RE*NiO_3_, as demonstrated in Fig. S16. Representatively, the resistance of the Pt-patterned NdNiO_3_ is elevated abruptly by ∼4 orders upon hydrogenation, while its resistances before and after hydrogenation were both within the range of ∼10^0^–10^4^ Ω (see Fig. S16). Thus, our LPE strategy also enables the optimum device resistance for the Mottronic functionalities of *RE*NiO_3_ catering for their reported analog electronic applications, such as artificial neuron and synapse devices [2,25].

### Providing alternative strategy for manufacturing analog discrete devices

Our LPE strategy further provides an alternative avenue to manufacture miniaturized discrete devices via encapsulating the micrometer-thick correlated perovskites over external oxide supporters, instead of directly using their bulk pellets. The perovskite encapsulations (perovskites@oxides) provide an alternative avenue for their applications in discrete devices, by separating the electronic and mechanical functions as provided by the encapsulation layer and supporter, respectively. This well circumvents the inherent high fragility and low resistivity of correlated perovskites, which are two major obstacles impeding their practical discrete device applications [20].

**Figure 3.**
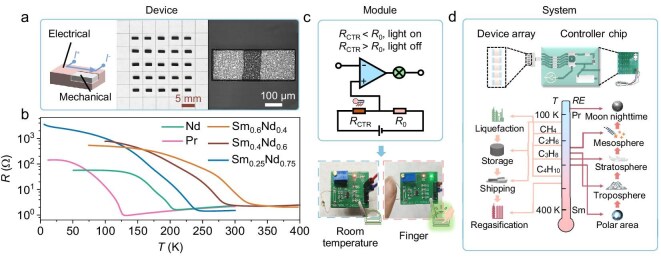
An alternative avenue for applying *RE*NiO_3_ in miniaturized analog discrete devices. (a) Illustration and images for as-made miniaturized CTR analog devices via directly encapsulating *RE*NiO_3_ on single-crystalline LaAlO_3_ supporter, giving rise to the separation of electronic and mechanical functionalities. (b) The temperature-dependent CTR device resistance based on *RE*NiO_3_ (e.g. *RE*: Sm_0.6_Nd_0.4_, Sm_0.4_Nd_0.6_, Sm_0.25_Nd_0.75_, Nd, and Pr). (c) Combining as-made CTR analog devices with a comparator circuit as temperature alarming modules, e.g. alarming the body temperature. (d) Combining alarming modules based on *RE*NiO_3_ with various *RE* to establish an array-like temperature alarming systems covering cryogenic ranges for applications, such as LNG industry and the aerospace engineering.

Accordingly, a set of CTR thermistor devices were fabricated via encapsulating miniature-sized single-crystalline LaAlO_3_ with oriented *RE*NiO_3_ (e.g. *RE*: Sm_0.6_Nd_0.4_, Sm_0.4_Nd_0.6_, Sm_0.25_Nd_0.75_, Nd, and Pr) within the KCl molten salt. Their representative images and *R–T* tendencies are demonstrated in Fig. 3a and b, respectively. Abrupt resistive switches are observed for as-made CTR thermistors at a critical temperature (*T*_C_) within 120–300 K at analog-compatible device resistances.

As-made CTR devices based on *RE*NiO_3_ encapsulations largely extend the range of *T*_C_ down to cryogenic temperature ranges, as yet uncoverable via mature CTR thermistors based on vanadium oxides [36] or doped BaTiO_3_ [37], as a more direct comparison provided in Fig. S17. To build a temperature alarming module for a specific critical temperature (*T*_C_), as-made CTR thermistors based on *RE*NiO_3_ encapsulations were connected to a comparator circuit. Representative modules alarming body temperature, sublimation point of dry ice, and gaseous point of liquid nitrogen are demonstrated in Fig. 3c and Fig. S18a (see also Video S1). Integrating such modules with various *T*_MIT_ establishes a temperature alarming array that can alarm a range of *T*_C_ with similar intervals, as illustrated in Fig. 3d. Compared to conventional cryogenic temperature sensing systems based on Pt sensors and analog-to-digital converters [38], our system exhibits one order of magnitude higher sensitivity and two orders of magnitude lower energy consumption, as further discussed in Fig. S18b. It well caters to the extensive application demands for temperature alarming in comprehensive cryogenic fields, such as the liquefied natural gas (LNG) industry [39], cryopreservation of cells and biologics [40], and aerospace-enabled aviation exploration [38].

### Further enabling wafer-scale growth for prospective IC applications

**Figure 4.**
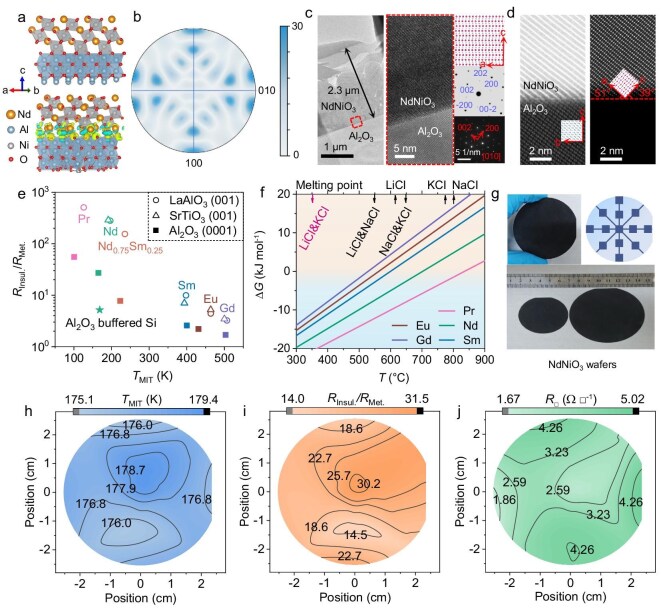
Realization of wafer-scale epitaxy of *RE*NiO_3_ for prospective integrated circuit applications. (a) The differential charge density for the NdNiO_3_/Al_2_O_3_ (0001) heterostructure from first-principles DFT calculations. (b) The pole figure for as-grown NdNiO_3_/Al_2_O_3_ (0001), derived from their XRD pattern using the GSAS. (c) The HAADF cross-section morphologies and selected area electron diffraction patterns for as-grown NdNiO_3_/Al_2_O_3_ (0001), indicating a micrometer-thick deposition with preferential orientation. (d) The crystallographic relationship between NdNiO_3_ and Al_2_O_3_ determined from HAADF images. See more details in Fig. S21. (e) Comparison of resistive switch (*R*_Insul._/*R*_Met._) across various critical temperatures (*T*_MIT_) for as-grown *RE*NiO_3_ on Al_2_O_3_ (0001), LaAlO_3_ (001), SrTiO_3_ (001), and Al_2_O_3_-buffered Si. More details are provided in Figs S22 and S23. (f) Formation free energy (Δ*G*) of *RE*NiO_3_ under ambient pressure plotted as a function of temperature. The MIT behaviors of as-grown *RE*NiO_3_ on sapphire in air or under flowing oxygen are shown in Fig. S25. (g) Representative images of the as-grown 2- and 3-inch NdNiO_3_/Al_2_O_3_ (0001). The schematic illustration indicates the regions, from which small pieces of samples were taken for further characterizations in order to evaluate the homogeneity in MIT functionality of the wafer. Wafer-scale mapping of the MIT properties for as-grown 2-inch NdNiO_3_ film: (h) the *T*_MIT_, (i) the *R*_Insul._/*R*_Met._, and (j) the sheet resistance (*R*_□_) at room temperature.

To further cater to prospective IC analog electronic applications, the *RE*NiO_3_ must be grown at wafer size on scalable substrates with potential lattice coherence at ‌high-index crystallographic planes. It is worth noting that the sapphire substrate can be easily fabricated at 2–6 inch wafer sizes, as were widely used in semiconductor engineering, such as GaN [41], SiC [42], and HgCdTe [43]. We performed the first-principles density functional theory (DFT) calculations to estimate the interfacial energy response for potential coherency between NdNiO_3_ (101) and Al_2_O_3_ (0001). The interface energy is calculated as *E* = (*E*_interface_ − *E*_NNO_ − *E*_AO_)/*S*, where *E*_interface_, *E*_NNO_, and *E*_AO_ are the total energies of the interface structure, NdNiO_3_, and Al_2_O_3_, respectively, while *S* is the interfacial area. The calculated interface energy is −0.258 eV/Å^2^ (see more details in Fig. S19a), the magnitude of which is similarly low compared to NdNiO_3_/LaAlO_3_ (−0.217 eV/Å^2^). The magnitude of charge density at the NdNiO_3_/Al_2_O_3_ interface is substantially larger than that of NdNiO_3_/LaAlO_3_, as shown in Fig. 4a and Fig. S19b, respectively, suggesting the thermodynamic driving force to achieve oriented growth of nickelate films on sapphire.

Based on the DFT estimations, we successfully grew *RE*NiO_3_ covering various rare-earth compositions on sapphire, as their representative XRD patterns shown in Fig. S20. The preferential crystal orientation for as-grown NdNiO_3_ on sapphire is indicated by the pole figure as derived by general structure analysis system (GSAS), as shown in Fig. 4b. Figure 4c and d further shows the cross-section morphologies of as-grown NdNiO_3_/Al_2_O_3_ (0001). As-grown NdNiO_3_ is of ∼2.3 μm thickness (see Fig. 4c) with a preferential (101) orientation relative to the Al_2_O_3_ (0001), as the epitaxy relationship more clearly illustrated in Fig. 4d. In addition to sapphire, successful growths of polycrystalline *RE*NiO_3_ showing abrupt MIT properties were also achieved on the Al_2_O_3_-buffered silicon substrate, via our LPE strategy (see Fig. S20). In Fig. 4e, we compare the MIT properties of *RE*NiO_3_ grown on Al_2_O_3_ to the ones on LaAlO_3_, via plotting their *R*_Insul._/*R*_Met_–*T*_MIT_. Their respective *R*_□_–*T* and TCR–*T* tendencies are shown in Fig. S22 for *RE*NiO_3_/Al_2_O_3_. Abrupt MIT functionalities were achievable for *RE*NiO_3_ grown on Al_2_O_3_, despite its smaller resistive switch compared to that grown on LaAlO_3_ and SrTiO_3_. In addition, similar Mottronic functionality as triggered by reversible protonation was also achieved in as-grown NdNiO_3_/Al_2_O_3_/Si, as more details demonstrated in Fig. S24.

To amplify the growth of *RE*NiO_3_ toward wafer scale, it is further necessary to stabilize their metastable perovskite structure in ambient oxygen or even in air, relieving their previous reliance on MPa-high *p*_O2_. According to the thermodynamic estimation shown in Fig. 4f, ambient pressure would provide sufficient *p*_O2_ for growing *RE*NiO_3_ (e.g. *RE* = Pr, Nd, Sm, Eu, and Gd), if the melting point of molten salt can be further descended (e.g. ∼350°C). Therefore, we used eutectic KCl and LiCl molten salt to grow *RE*NiO_3_ at 500–700 °C in air or flowing oxygen, in which situation abrupt MIT properties similar to the MPa-high *p*_O2_ growth were achieved for *RE*NiO_3_ with a variety of *RE* including Pr, Nd, Sm, Eu, and Gd.

Hence, it is capable of amplifying the growth of *RE*NiO_3_ on 2- and 3-inch sapphire wafers, as their representative images shown in Fig. 4g. The uniformity of MIT for as-grown *RE*NiO_3_ wafers was benchmarked via measuring their *R*_□_–*T* at multiple positions [36], as marked in Fig. 4g. Figure S26 shows their respective *R*_□_–*T* tendencies for the 2-inch NdNiO_3_ and PrNiO_3_. Accordingly, their *T*_MIT_, resistive switch, and the magnitude of *R*_□_ at room temperature were calculated and shown in Fig. 4h–j, respectively. The uniformity of the MIT properties is indicated by the low standard deviation (s.d.) in their *T*_MIT_ (e.g. 0.99 K for NdNiO_3_ and 1.2 K for PrNiO_3_) and *R*_□_ (e.g. 0.9 Ω/□ for NdNiO_3_ and 1.0 Ω/□ for PrNiO_3_), while abrupt resistive switch was achieved over ∼80% of the wafer area. Enabling the scalable growth of *RE*NiO_3_ at micrometer-thick further paves the way to their practical applications in the integrated circuit electronics.

### Extendibility to the growth of other functional oxide systems

In addition to nickelates, our LPE strategy was also successfully extended to other multicomponent oxides, such as Pr_0.67_Sr_0.33_MnO_3_, SrRuO_3_, and Bi_2_Sr_2_CaCu_2_O_8_ (see Fig. S27). Thus, it exhibits broad applicability in the micrometer-thick film growth of both perovskites and non-perovskites correlated systems, covering a wealth of functionalities, such as magnetoresistance, oxide electrode, and superconductivity.

## CONCLUSION

To conclude, we have opened up a molten-salt-assisted avenue enabling effective and scalable growth of correlated perovskites with micrometer-thick preferential orientation maintained. The ultra-stable thermodynamic environment and consistent precursor availability, as provided within molten alkali-metal chlorides, sustain long-period oriented epitaxy, counteracting inherent metastability and/or lattice mismatch. It well addresses the technological discontinuity in micrometer-thick growth of correlated perovskites even at thermodynamic metastability, such as *RE*NiO_3_, enabling their analog-compatible device resistance and record-competing electronic functionalities. From the one side, encapsulating *RE*NiO_3_ over external oxide supporters via LPE provides an alternative way to manufacture discrete devices at miniaturized sizes, circumventing their inherent fragility. Accordingly, the *RE*NiO_3_-based cryogenic CTR device, module, and systems were developed, effectively filling the present technological void for a quantum leap in sensitivity and energy efficiency in cryogenic alarming. From the other side, wafer-scale epitaxy of the metastable *RE*NiO_3_ on 2-inch sapphire with high uniformity in *T*_MIT_ (e.g. s.d. of ∼1 K) was achieved via low-temperature LPE within eutectic molten salts, paving the way to viable utilization in analog IC electronics. Our strategy is easily extendable to other correlated oxides systems, providing a general gateway to bridge gaps between their fundamental frontier and practical analog electronic applications.

## METHODS

### *RE*NiO_3_ film growth

The *RE*NiO_3_ films were grown using an alkali-metal halide molten-salts assisted LPE strategy. Oxide precursors, e.g. *RE*_2_O_3_ and NiO, were stoichiometrically mixed together with the alkali metal halide molten salts, such as KCl, LiCl, NaCl, and their mixtures, and afterward pressed on the surface of a substrate within a crucible. The crucible was firstly annealed at 50–150°C above the melting point of molten salts, followed by slowly cooling down. The *RE*NiO_3_ exhibiting light-*RE* (e.g. *RE* = Pr and Nd) were grown within KCl molten salt at 600–900°C under 7 MPa *p*_O2_, or the KCl and LiCl eutectic molten salt at 500–800°C in air. The *RE*NiO_3_ with middle-*RE* (e.g. *RE* = Sm, Eu and Gd) were grown under a higher *p*_O2_ of 10 MPa, or KCl and LiCl eutectic molten salt at 500–800°C in flowing oxygen.

### Fabrication of *RE*NiO_3_ encapsulates and CTR devices

To encapsulate *RE*NiO_3_ on supporting oxides, we pressed cuboid-shaped single-crystalline oxides (e.g. LAO, STO, LSAT, or sapphire) together with the oxide precursors (e.g. *RE*_2_O_3_ and NiO) and alkali metal halides molten salt of KCl within a crucible. Afterward, a series of annealing processes at 7–10 MPa *p*_O_2__ were performed first at 900°C and further at 700–800 °C, followed by slowly cooling down to the room temperature for encapsulating *RE*NiO_3_ on the supporting oxides. To further make the CTR thermistor based on the *RE*NiO_3_ encapsulates, Pt was sputtered on the two ends as contacting electrode. The as-made CTR thermistors were further connected with a comparator circuit using TL331 comparator to build a temperature alarming module.

### Characterization

The crystal structures of as-grown *RE*NiO_3_ were characterized by XRD analysis using a Bruker (D8, Advance, Germany). The cross-section morphologies were characterized via the scanning electron microscope (SEM) and scanning transmission electron microscopy (STEM) performed by an FEI Titan Themis Z microscope. The temperature dependences of sheet resistances were measured by the physical property measurement system within the temperature range from 10 to 400 K, and the constant temperature anemometer within the temperature range from 300 to 550 K. The electronic structures were probed by the NEXAFS analysis at the 4B9B beamline in Beijing Synchrotron Radiation Facility, Institute of High Energy Physics, Chinese Academy of Sciences. XPS (Thermo Scientific ESCALAB 250Xi) was used to exclude contaminations by molten salt.

### Theoretical calculation

All calculations were performed using DFT within the Vienna *ab Initio* Simulation Package [44]. Exchange–correlation effects were treated within the generalized gradient approximation [45] using the Perdew–Burke–Ernzerhof revised for solids functional [46]. A plane-wave kinetic energy cutoff of 500 eV was adopted, and projector augmented-wave (PAW) pseudopotentials were employed. The Monkhorst–Pack *k*-point mesh was set to 5 × 5 × 1, and a 20 Å vacuum region along the *c*-direction was adopted to prevent periodic interactions. The in-plane lattice constant was fixed to *a* = *b* = 9.5160 Å and *γ* = 120° (the lattice constant of Al_2_O_3_ substrate), and the atomic positions were fully relaxed.

## Supplementary Material

nwag216_Supplemental_File
