# Supplementary material for: A scalable molten-salt epitaxy of analog-compatible correlated perovskites at micrometer-scale thickness
Source: Natl Sci Rev. 2026 Apr 8;13(10):nwag216. doi: 10.1093/nsr/nwag216 (PMC13234942; doi:10.1093/nsr/nwag216)
Supplement: nwag216_Supplemental_File [file nwag216_supplemental_file.pdf]

Supplementary information for:

A scalable molten-salt epitaxy of analog-compatible correlated-perovskites at μm-thick

Yi Bian (边驿)[1,†], Peiheng Jiang (蒋沛恒)[2,†], Nuofu Chen (陈诺夫)[3], Hao Zhang (张豪)[1], Binghui Ge (葛炳辉)[4,*], Hongliang Dong (董洪亮)[5], Jiaou Wang (王嘉鸥)[6], Ho-kwang Mao (毛河光)[5], Lidong Chen (陈立东)[7,*] and Jikun Chen (陈吉堃)[1,*]

[1]Beijing Advanced Innovation Center for Materials Genome Engineering, School of Materials Science and Engineering, University of Science and Technology Beijing, Beijing 100083, China;

[2]School of Physics, MOE Key Laboratory for Nonequilibrium Synthesis and Modulation of Condensed Matter, Xi'an Jiaotong University, Xi'an 710049, China;

[3]School of New Energy, North China Electric Power University, Beijing 102206, China;

[4]State Key Laboratory of Opto-Electronic Information Acquisition and Protection Technology, Institute of Physical Science and Information Technology, Anhui University, Hefei 230601, China;

[5]Center for High Pressure Science and Technology Advanced Research, Shanghai 201203, China;

[6]Beijing Synchrotron Radiation Facility, Institute of High Energy Physics, Chinese Academy of Sciences, Beijing 100049, China;

[7]Shanghai Institute of Ceramics, Chinese Academy of Sciences, Shanghai 200050, China

***Corresponding authors**. E-mails: jikunchen@ustb.edu.cn; cld@mail.sic.ac.cn; bhge@ahu.edu.cn

[†]Equally contributed to this work.

Section 1. Thermodynamic calculation for synthesizing *RE*NiO$_3$

In section 1, more details are shown for the calculation of Gibbs free energy (ΔG) for synthesizing *RE*NiO$_3$ with various rare-earth composition at different temperatures and oxygen partial pressures. Fig. S1 shows the ΔG of *RE*NiO$_3$ in air and 7 MPa oxygen pressure, plotted as a function of the ionic rare-earth radius.

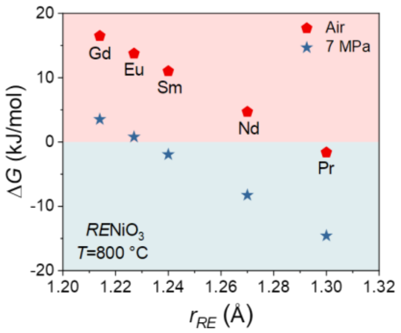

Fig. S1 | Thermodynamic stability of *RE*NiO$_3$ in air and 7 MPa oxygen pressure at 800°C. The calculated formation Gibbs free energy (ΔG) of *RE*NiO$_3$ plotted as a function of the ionic radius of the rare-earth (r_{RE}) in air and 7 MPa oxygen pressure at 800°C. The chemical reaction of synthesizing *RE*NiO$_3$ is represented by:

$$\frac{1}{2}RE_2O_3 + NiO + \frac{1}{4}O_2 = RENiO_3 \tag{1}$$

The Gibbs free energy was calculated according to the formula [1],

$$\Delta G = \Delta H_{\mathrm{LNO}} - T\Delta S_{\mathrm{LNO}} + (c + dT)\big(r(RE^{3+}) - r(La^{3+})\big) - \frac{1}{4}RTln(p_{O_2}) \tag{2}$$

where ΔH_{LNO} and ΔS_{LNO} are the enthalpy and entropy of preparing LaNiO$_3$ at 1000 K, respectively. c and d are thermodynamic coefficients, R is the ideal gas constant, p_{O2} is the oxygen pressure, and $r(RE^{3+})$ is the rare-earth ionic radius. c and d were obtained via critical synthesis conditions for *RE*NiO$_3$ within various rare-earth compositions [2, 3]. Table S1 further provides the magnitudes of the above thermodynamic parameters and ionic rare-earth radius. The reduction of Gibbs free energy can be achieved through either lowering the system temperature or elevating the oxygen partial pressure.

Under equilibrium conditions, the relationship between T and p_{O2} follows as

$$log(p_{O_2}) = \frac{4}{ln10 \times RT}\left[\Delta H_{\mathrm{LNO}} - T\Delta S_{\mathrm{LNO}} + (c + dT)\big(r(RE^{3+}) - r(La^{3+})\big)\right] \tag{3}$$

It can be seen that the decreasing in r_{RE} elevates ΔG towards the positive magnitude, hence *RE*NiO$_3$ exhibiting heavier *RE* is more difficult to be synthesized.

Table S1| The magnitudes in the thermodynamic parameters [1–3] and ionic radius of rare-earth [4] as used in the calculations

Parameter	Value
c	20.198 kJ mol^{-1} Å^{-1}
d	-0.215 kJ K^{-1} mol^{-1} Å^{-1}
ΔH_{LNO}	-46.07 kJ mol^{-1}
ΔS_{LNO}	-2.64×10^{-2} kJ (K^{-1} mol^{-1})
$r(La^{3+})$	1.36 Å
$r(Pr^{3+})$	1.30 Å
$r(Nd^{3+})$	1.27 Å
$r(Sm^{3+})$	1.24 Å
$r(Eu^{3+})$	1.227 Å
$r(Gd^{3+})$	1.214 Å

Section 2. The crystal structure, morphology and electronic structure of *RE*NiO_3 films on perovskite substrate.

In section 2, more results are provided for the crystal structure, morphology and electronic structure for *RE*NiO_3 grown on single crystalline perovskite oxide substrates, such as $LaAlO_3$ (LAO), $SrTiO_3$ (STO) and $(LaAlO_3)_{0.3}(Sr_2AlTaO_6)_{0.7}$ (LSAT), at orientations of (001), (110) and (111). Fig. S2 shows more cross section morphologies of $SmNiO_3$ grown on $LaAlO_3$ and $SrTiO_3$ substrates with various orientations. Fig. S3 shows the X-ray diffraction patterns of $NdNiO_3$ grown on various substrates and orientations. Fig. S4 shows the estimated lattice relaxation rate of $NdNiO_3$ across the material thickness from reciprocal space mappings. Fig. S5 shows the X-ray photoelectron spectroscopy analysis for representative *RE*NiO_3 films. Fig. S6 shows elements distribution of *RE*NiO_3 grown on $LaAlO_3$ and $SrTiO_3$ substrates. Fig. S7 shows the cross-section morphologies of *RE*NiO_3 films grown on $SrTiO_3$ (001). Fig. S8 shows the current versus electric-field characteristics measured for as-grown $SmNiO_3$ film. Fig. S9 shows the near edge X-ray absorption fine structure analysis for *RE*NiO_3 films grown on $LaAlO_3$ (001).

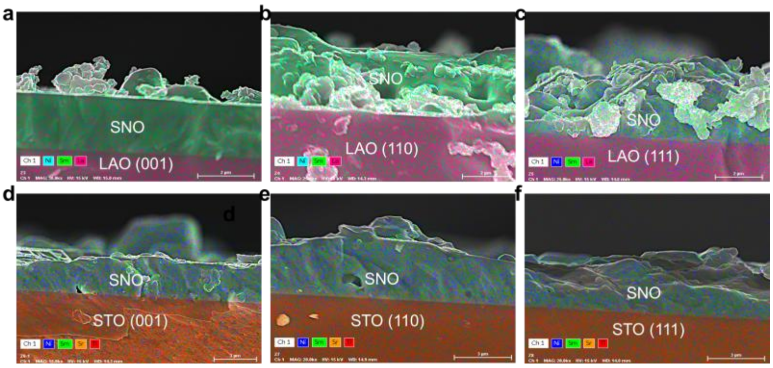

Fig. S2 | Cross-section morphology of the as-grown $SmNiO_3$ films. The energy dispersive spectroscopy mapping of $SmNiO_3$ (SNO) films on $LaAlO_3$ (LAO) (a–c), and $SrTiO_3$ (STO) (d–f), with (001), (110) and (111) orientations. These results demonstrate that as-grown *RE*NiO_3 exhibit thickness in micrometer-scale.

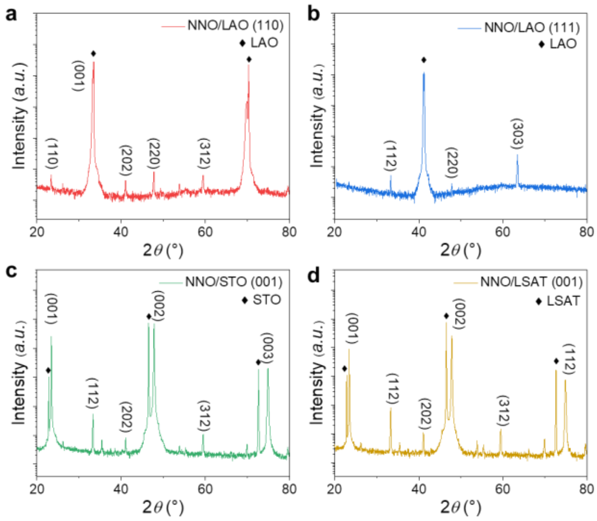

Fig. S3 | Additional X-ray diffraction (XRD) patterns of $NdNiO_3$ (NNO) films grown on crystalline perovskite substrate. (a), $LaAlO_3$ (LAO) (110), (b), LAO (111), (c), $SrTiO_3$ (STO) (001) and (d), $(LaAlO_3)_{0.3}(Sr_2AlTaO_6)_{0.7}$ (LSAT) (001). The film peaks adjacent to the substrate displays higher intensity compared to other ones, indicating the preferentially orientated growth of $NdNiO_3$ on these single crystal perovskites substrates at various crystal orientations.

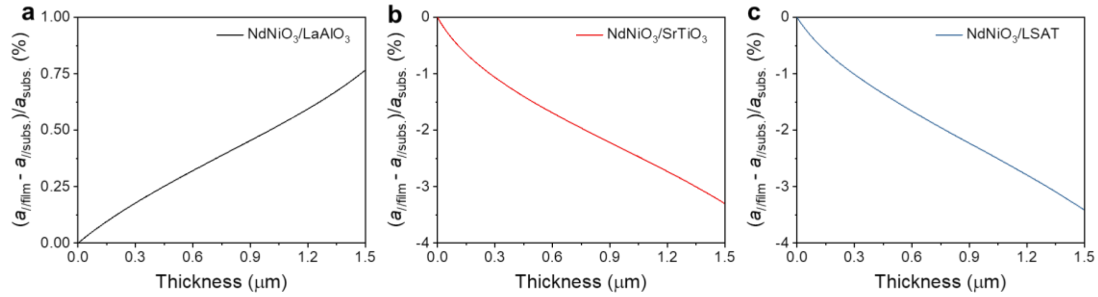

Fig. S4 | The estimated lattice relaxation rate of $NdNiO_3$ across the material thickness from reciprocal space mappings shown in Fig. 2c. The in-plane lattice relaxation ratio, e.g., ($a_{//,\mathrm{film}}$ - $a_{//,\mathrm{sub.}}$)/ $a_{//,\mathrm{sub.}}$, plotted as a function of material thickness for $NdNiO_3$ films grown on various substrates with (001) orientation. (a), $NdNiO_3/LaAlO_3$. (b), $NdNiO_3/SrTiO_3$. (c), $NdNiO_3/(LaAlO_3)_{0.3}(Sr_2AlTaO_6)_{0.7}$ (LSAT).

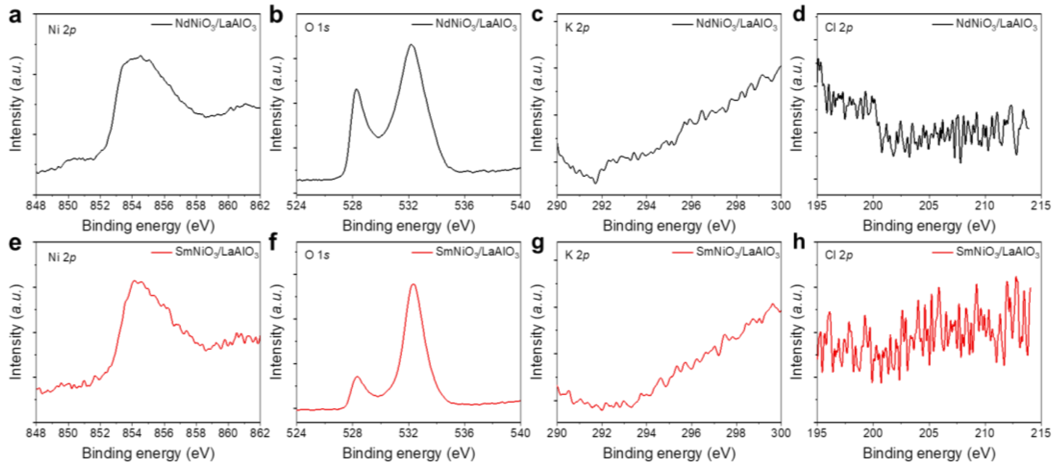

Fig. S5 | The X-ray photoelectron spectroscopy (XPS) analysis for representative $\textit{RE}NiO_3$ films to exclude contaminations by the molten salt. (a–d), The XPS spectra of $NdNiO_3/LaAlO_3$ film. (a), Ni 2*p*. (b), O 1*s*. (c), K 2*p*. (d), Cl 2*p*. (e–h) The XPS spectra of $SmNiO_3/LaAlO_3$ film. (e), Ni 2*p*. (f), O 1*s*. (g), K 2*p*. (h) Cl 2*p*. The spectra of Ni of Ni 2*p* and O 1*s* show similar features with the previous literatures [5]. The absence of K 2*p* and Cl 2*p* signals in both $NdNiO_3$ and $SmNiO_3$ films indicates negligible contamination by the molten salts during the growth process.

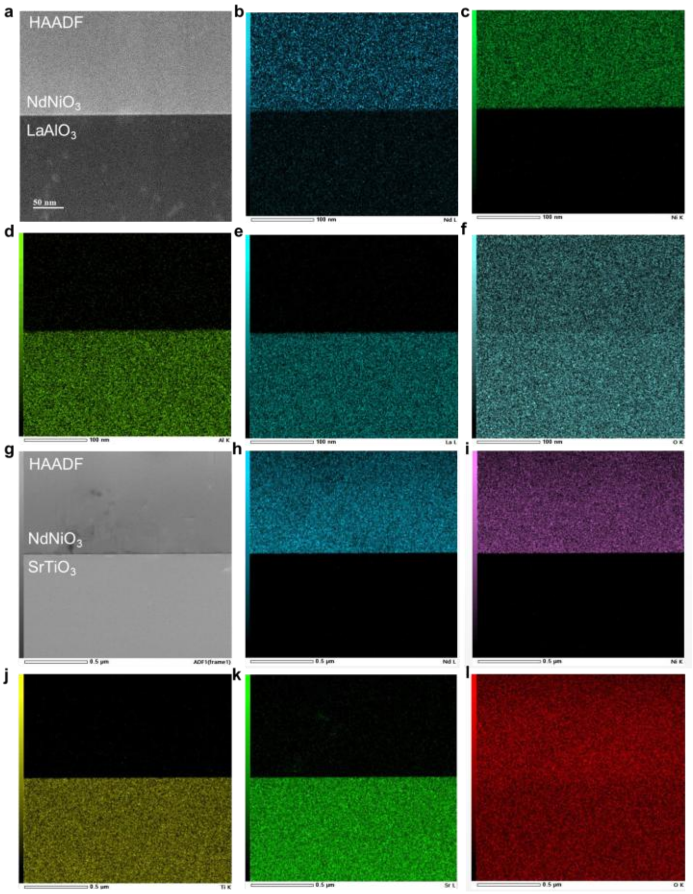

Fig. S6 | Elemental distribution of the as-grown $NdNiO_3/LaAlO_3$ (001) and $NdNiO_3/SrTiO_3$ (001) films. (a), The cross-sectional high-angle annular dark-field (HAADF) image of $NdNiO_3/LaAlO_3$. (b–f), The Energy Dispersive Spectroscopy (EDS) mapping of (b) Nd, (c) Ni, (d) Al, (e) La, and (f) O for $NdNiO_3/LaAlO_3$, respectively. (g), The cross-sectional HAADF image of $NdNiO_3/SrTiO_3$. (h–l), The EDS mapping of (h) Nd, (i) Ni, (j) Ti, (k) Sr, and (l) O for $NdNiO_3/SrTiO_3$, respectively. These results demonstrate that there is no elementary diffusion between film and substrate at their interface for as-grown $NdNiO_3/LaAlO_3$ and $NdNiO_3/SrTiO_3$.

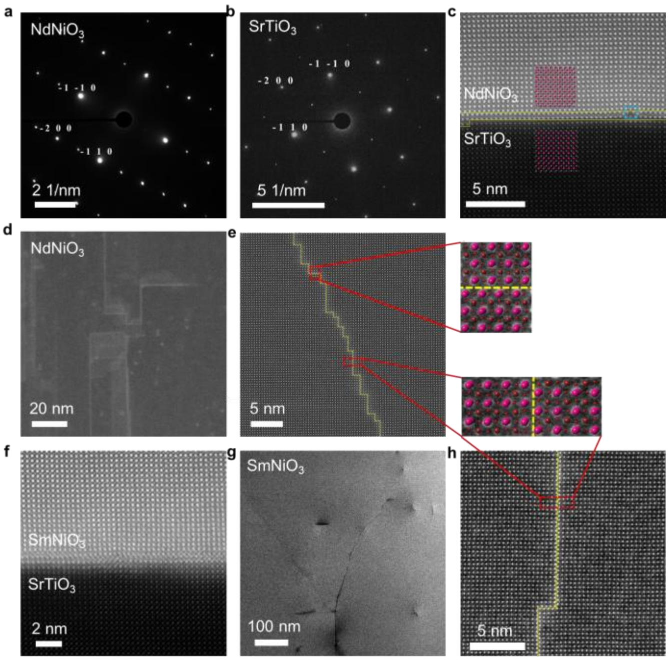

Fig. S7 | Cross-section morphologies of *RE*NiO$_3$ films grown on SrTiO$_3$ (001). (a–b), The selected area electron diffraction (SAED) patterns of NdNiO$_3$/SrTiO$_3$ (001) for: (a), the films and (b), the substrate. (c–e) High-angle annular dark-field (HAADF) morphologies of NdNiO$_3$/SrTiO$_3$ (001). (c), The cross-section morphology NdNiO$_3$/SrTiO$_3$ (001), where the vacancies and the stacking faults are marked by the blue square and yellow dash-lines, respectively. The lattice mismatch between NdNiO$_3$ and SrTiO$_3$ relaxed by the interfacial stacking faults. (d), Stacking faults observed within the films that relax the interfacial strains. (e), Representative morphology of the displacement between adjacent rock-salt layers. (f–h), HAADF morphologies of SmNiO$_3$/SrTiO$_3$ (001). (f), The interfacial cross-section morphology for as-grown SmNiO$_3$/SrTiO$_3$ (001). (g), The stacking faults observed within the SmNiO$_3$ film at a low magnification. (h), The displacement between adjacent rock-salt layers observed at a high magnification. Summarizing above results, it can be seen that both SmNiO$_3$ and NdNiO$_3$ were well grown orientally on SrTiO$_3$ substrate via our molten salt assisted liquid phase epitaxy strategy, despite a larger lattice mismatch compared to their analogous growth on LaAlO$_3$ (see Fig. 2d–2h). This is attributed to the effective formation of stacking faults at both the interface and within the film that mildly and gradually transform the film lattice constant from the magnitude same as the substrate towards their bulk.

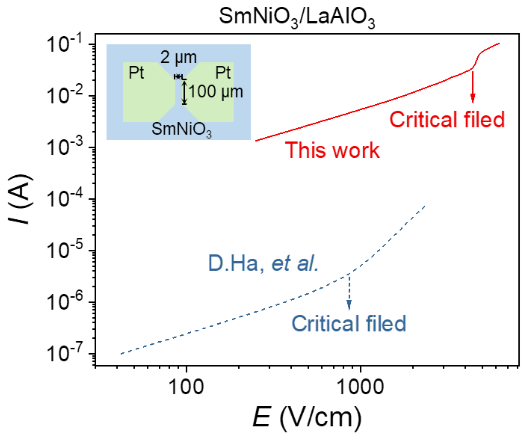

Fig. S8 | The current versus electric-field characteristics measured for as-grown $SmNiO_3$ films compared to the literature [6]. As illustrated by the inset, two Pt electrodes were patterned on the surface of as-grown $SmNiO_3$ film, from where the electric voltage was imparted. At the beginning, the current between the two Pt electrode increases linearly with an enlarging of the imparted voltage (electric field), until reaching a critical magnitude to trigger the metal-insulator transition (MIT). The MIT drives the electronic structure of $SmNiO_3$ from semiconductor to metallic phase, which reduces its resistivity abruptly, so that an abrupt elevation in the current is observed by further enlarging electric field beyond the critical point. Compared to the previous report [6], a larger magnitude in the critical field triggering MIT (or transition from Ohmic conduction to space-charge limited conduction) is observed for the presently grown sample. This implies a higher crystallinity free of defect, which may contribute to leakage. Also, the much larger magnitude in current is in agreement to the presently much higher thickness, compared to the previous growth.

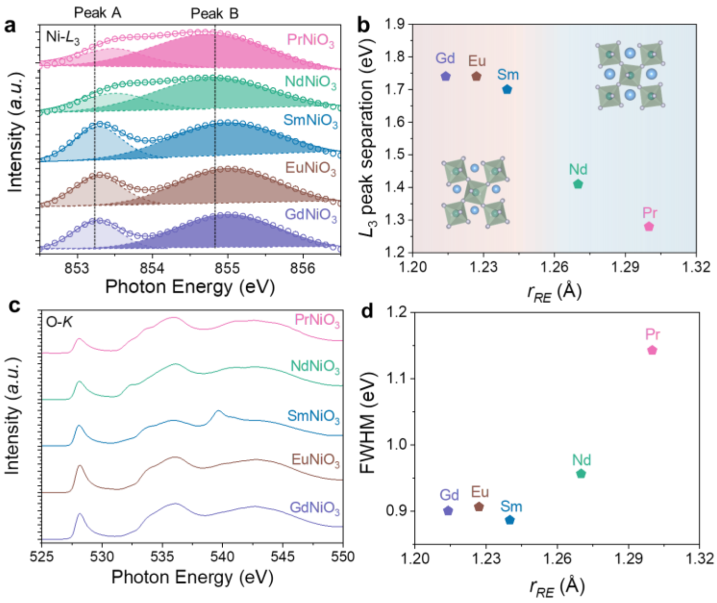

Fig. S9 | The near edge X-ray absorption fine structure (NEXAFS) results for $RENiO_3/LaAlO_3$ (001) films. (a), The NEXAFS spectra of the Ni-L_3 edge for as-grown $RENiO_3$ with various *RE*. (b), The magnitude in split within Ni-L_3 edge for $RENiO_3$ plotted as a function of their ionic rare-earth radius (r_{RE}). (c), The NEXAFS spectra of the O-*K* edge for as-grown $RENiO_3$ with various *RE*. (d), The full width at half maxima (FWHM) of the O-*K* pre-peak. A more pronounced separation in the Ni-L_3 edge and smaller FWHM in the O-*K* pre-peak were observed for $RENiO_3$ with heavier *RE*, indicating their larger charge transfer gap and more strengthened insulating orbital configurations. These results confirm a strengthened electron localized electronic phase for $RENiO_3$ with heavier *RE* owing to the reduced orbital overlapping between Ni-3*d* and O-2*p*.

Section 3. Electrical transportations of *RE*NiO_3 films grown on perovskite substrate.

In section 3, more results are provided for the electrical transportation properties of *RE*NiO_3 grown on various types of single crystalline perovskite oxide substrates at more crystal orientations. Fig. S10 shows the determination of critical temperature associated with metal-insulator transition (T_{MIT}) of *RE*NiO_3/$LaAlO_3$ (001). Fig. S11 shows the room temperature resistivity achieved in the presently grown *RE*NiO_3 compared with the previous reports. Fig. S12 shows the determination of the resistive change across critical temperature ($R_{Insul.}/R_{Met.}$) of *RE*NiO_3/$LaAlO_3$ (001). Fig. S13 and Fig. S14 show the determination of T_{MIT} and $R_{Insul.}/R_{Met.}$ of *RE*NiO_3/$SrTiO_3$ (001). Fig. S15 shows the metal-insulator transition properties of *RE*NiO_3 grown on more types of substrates and orientations demonstrated by their *R-T* and *TCR-T* tendencies. Fig. S16 shows more results for the resistive change during the hydrogen annealing induced Mottronic transition of $NdNiO_3$/$LaAlO_3$ (001). Fig. S17 shows the temperature dependence in resistance for the as-made *RE*NiO_3@$LaAlO_3$ critical temperature resistance (CTR) thermistors compared with the reported ones based on VO_2-CTR, doped $BaTiO_3$ and Pt thermistors. Fig. S18 shows the images and superiority of as-made analog temperature alarming.

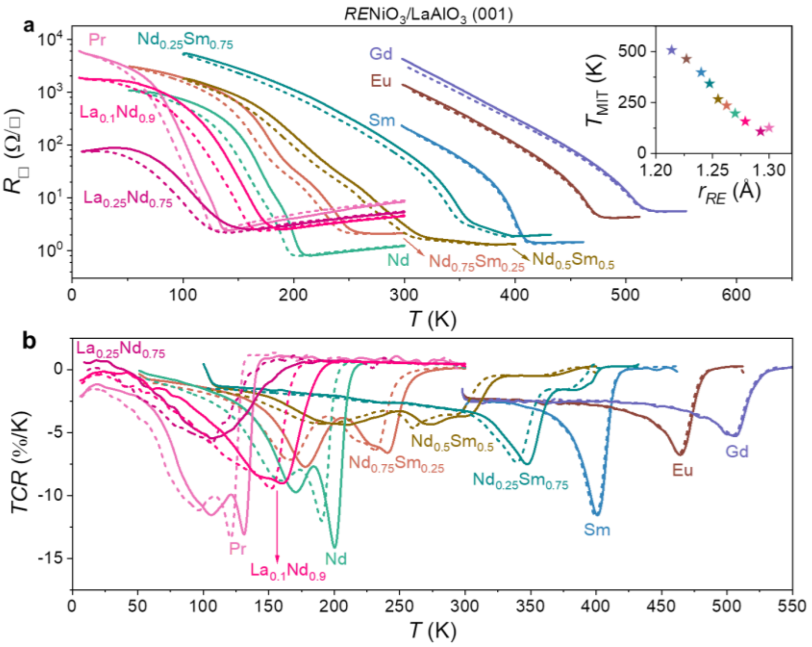

Fig. S10 | The metal-insulator transition behavior of *RE*NiO_3 films grown on $LaAlO_3$ (001) substrate. (a), The temperature dependent sheet resistances ($R_{\square}$) measured for the as-grown *RE*NiO_3. The inset shows the critical temperature (T_{MIT}) plotted as a function of the ionic rare-earth radius (r_{RE}). (b), The temperature coefficient of resistance (*TCR*) plotted as a function of temperature for the as-grown *RE*NiO_3. The solid and dashed lines show the heating and cooling processes, respectively. To determine T_{MIT}, we calculated the *TCR* as $TCR = (R_2 - R_1)/R_1/(T_2 - T_1)$. The minimum in the *TCR-T* tendency is considered as $T_{MIT,H}$ and $T_{MIT,C}$ for heating up and cooling down processes, respectively. The T_{MIT} was determined as the average magnitude of $T_{MIT,H}$ and $T_{MIT,C}$. The results indicate the widely tunable metal-insulator transition functionality of as-grown *RE*NiO_3 films on $LaAlO_3$ (001) via liquid phase epitaxy strategy.

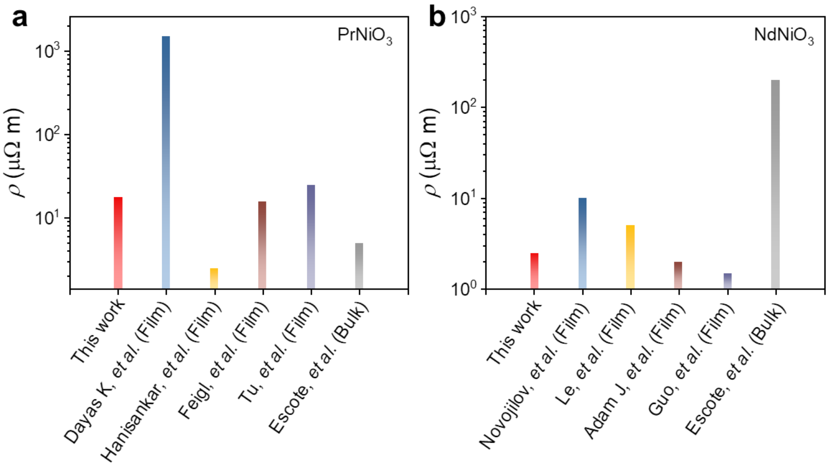

Fig. S11 | Comparing the room temperature resistivity achieved in the presently grown *RE*NiO_3 with the previous reports [3, 7–14]. (a) $PrNiO_3$. (b) $NdNiO_3$. It can be seen that the resistivity of as-grown $PrNiO_3$ and $NdNiO_3$ are within the range of reported magnitudes.

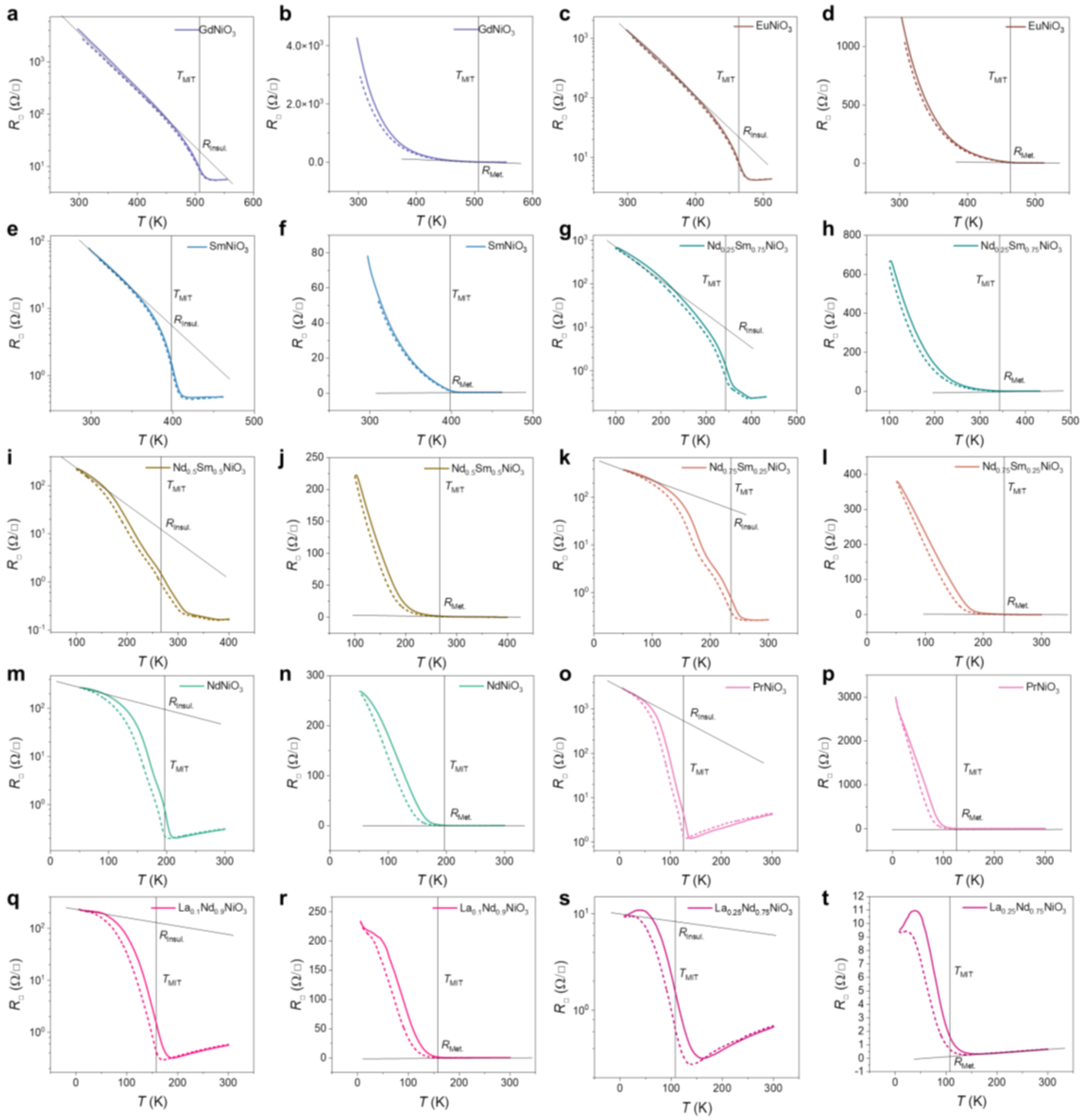

Fig. S12 | Determination of the resistive change across critical temperature ($R_{\mathrm{Insul.}}/R_{\mathrm{Met.}}$) of $RE\mathrm{NiO_3}/\mathrm{LaAlO_3}$ (001) films. (a–t), The $R_{\mathrm{Insul.}}$ and $R_{\mathrm{Met.}}$ across critical temperature associated with metal-insulator transition (T_{MIT}) of $RE\mathrm{NiO_3}/\mathrm{LaAlO_3}$ (001) films. (a) and (b), $GdNiO_3$. (c) and (d) $EuNiO_3$. (e) and (f) $SmNiO_3$. (g) and (h) $Nd_{0.25}Sm_{0.75}NiO_3$. (i) and (j) $Nd_{0.5}Sm_{0.5}NiO_3$. (k) and (l) $Nd_{0.75}Sm_{0.25}NiO_3$. (m) and (n) $NdNiO_3$. (o) and (p) $PrNiO_3$. (q) and (r) $La_{0.1}Nd_{0.9}NiO_3$. (s) and (t) $La_{0.25}Nd_{0.75}NiO_3$. To calculate the $R_{\mathrm{Insul.}}$ and $R_{\mathrm{Met.}}$, linear fitting was performed on the insulating and metallic phases on the sheet resistance-temperature ($R_{\square}$-T) tendencies. Subsequently, the sheet resistances at the intersection point of the two linear fits with T_{MIT} were considered as $R_{\mathrm{Insul.}}$ and $R_{\mathrm{Met.}}$, respectively. The $R_{\mathrm{Insul.}}/R_{\mathrm{Met.}}$ was determined as $R_{\mathrm{Insul.}}$ divided by $R_{\mathrm{Met.}}$.

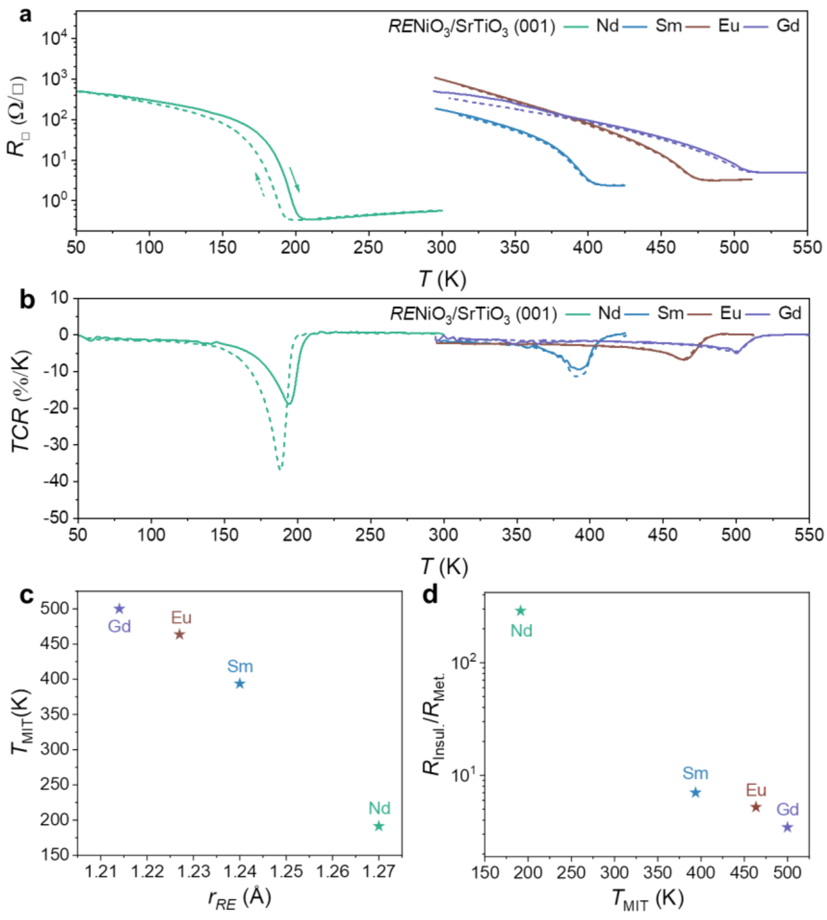

Fig. S13 | The metal-insulator transition behavior of *RE*NiO_3 films grown on $SrTiO_3$ (001) substrate. (a), The temperature dependent sheet resistances ($R_{□}$) measured for the as-grown *RE*$NiO_3/SrTiO_3$ (001), demonstrating their widely tunable metal-insulator transition (MIT) functionality. (b), The temperature coefficient of resistance (*TCR*) plotted as a function of temperature for the as-grown *RE*$NiO_3/SrTiO_3$ (001). To determine T_{MIT}, we calculated the *TCR* as $TCR = (R_2 - R_1)/R_1/(T_2 - T_1)$. The minimum in the *TCR*-*T* tendency is considered as $T_{MIT,H}$ and $T_{MIT,C}$ for heating up and cooling down processes, respectively. The T_{MIT} was determined as the average magnitude of $T_{MIT,H}$ and $T_{MIT,C}$. (c), The metal-insulator transition temperature (T_{MIT}) as a function of the radius of rare-earth (r_{RE}). (d), The resistive change across critical temperature ($R_{Insul.}/R_{Met.}$) plotted as a function of T_{MIT}. Similar abrupt MIT behaviors are achieved in as-grown *RE*$NiO_3/SrTiO_3$ compared to *RE*$NiO_3/LaAlO_3$ (see Extended Data Fig. 4), while an analog-compatible sheet resistance was also enabled.

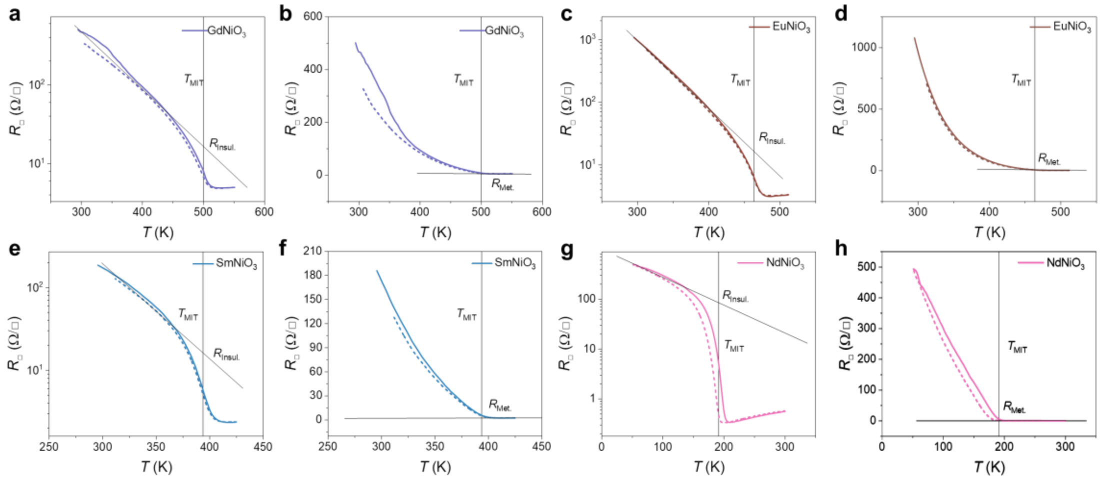

Fig. S14 | Determination of the resistive change across critical temperature ($R_{Insul.}/R_{Met.}$) of RENiO$_3$/SrTiO$_3$ (001) films. (a–h), The $R_{Insul.}$ and $R_{Met.}$ across critical temperature associated with metal-insulator transition (T_{MIT}) of RENiO$_3$/SrTiO$_3$ (001) films. (a) and (b), GdNiO$_3$. (c) and (d) EuNiO$_3$. (e) and (f) SmNiO$_3$. (g) and (h) NdNiO$_3$. To calculate the $R_{Insul.}$ and $R_{Met.}$, linear fitting was performed on the insulating and metallic phases on the sheet resistance-temperature ($R_{\square}$-T) tendencies. Subsequently, the sheet resistances at the intersection point of the two linear fits with T_{MIT} were considered as $R_{Insul.}$ and $R_{Met.}$, respectively. The $R_{Insul.}/R_{Met.}$ was determined as $R_{Insul.}$ divided by $R_{Met.}$.

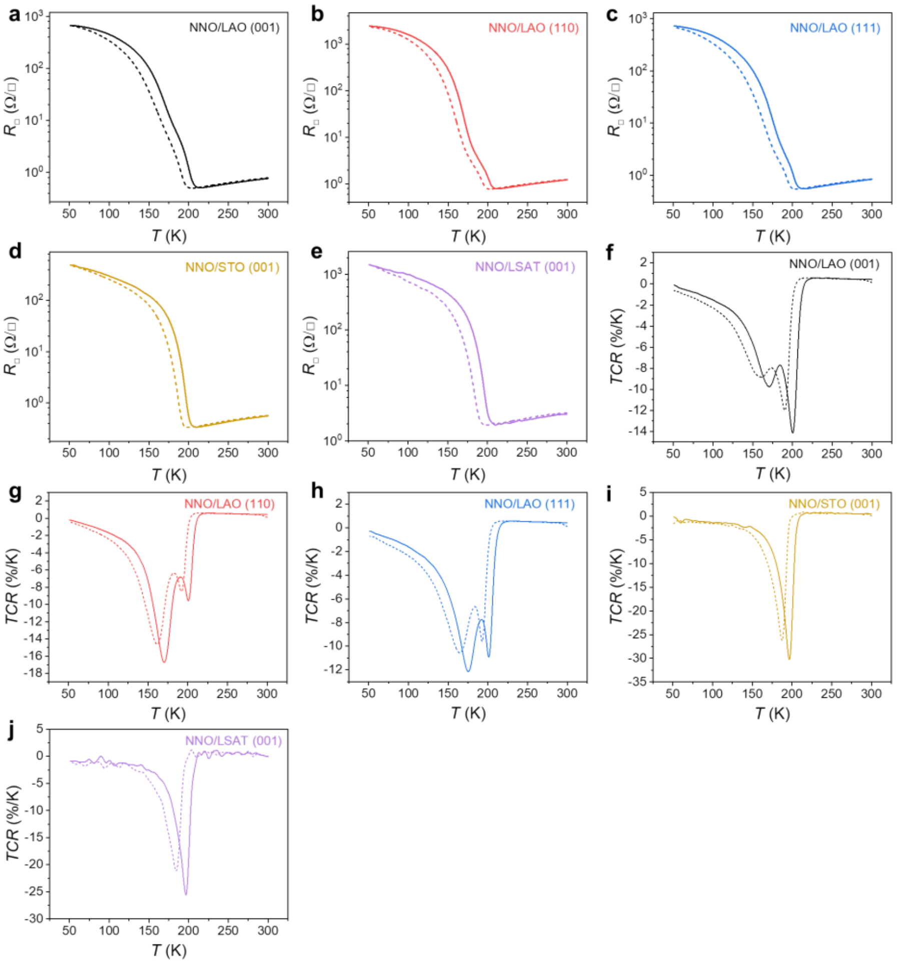

Fig. S15 | The electrical measurements of $NdNiO_3$ (NNO) films on various types and orientations of perovskite substrates. (a–e), The sheet resistance plotted as a function of temperature for NNO grown on various types and orientations of perovskite substrates. (a) NNO/$LaAlO_3$ (LAO) (001). (b) NNO/LAO (110). (c) NNO/LAO (111). (d) NNO/$SrTiO_3$ (STO) (001). (e) NNO/$(LaAlO_3)_{0.3}(Sr_2AlTaO_6)_{0.7}$ (LSAT) (001). (f–j), The temperature coefficient of resistance (*TCR*) plotted as a function of temperature for NNO grown on various types and orientations of perovskite substrates. (f) NNO/LAO (001). (g) NNO/LAO (110). (h) NNO/LAO (111). (i) NNO/STO (001). (j) NNO/LSAT (001). These results demonstrate the as-grown *RE*NiO_3 films on various types of perovskite substrates at varied orientations via molten-salt assisted liquid phase epitaxy strategy exhibit similar metal-insulator transition properties compared to the ones grown on LAO (001).

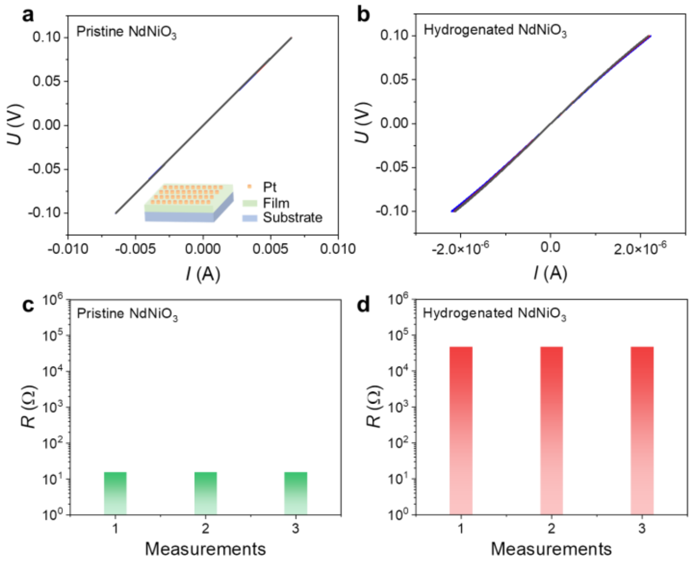

Fig. S16 | The Mottronic properties of $NdNiO_3/LaAlO_3$ (001). (a–b), The representative current-voltage (*I-V*) curve measured for (a) the pristine and (b) the hydrogenated $NdNiO_3$ with platinum patterns. The schematic illustration shows the platinum patterns used to trigger the hydrogen-induced electronic transition. (c–d), The resistances of $NdNiO_3$ with top platinum patterns (c) before and (d) after the hydrogenation process. The hydrogenation elevates the resistivity of $NdNiO_3$ by 3 orders, demonstrating the Mottronic functionality in the presently grown nickelates via molten-salt assisted liquid phase epitaxy strategy.

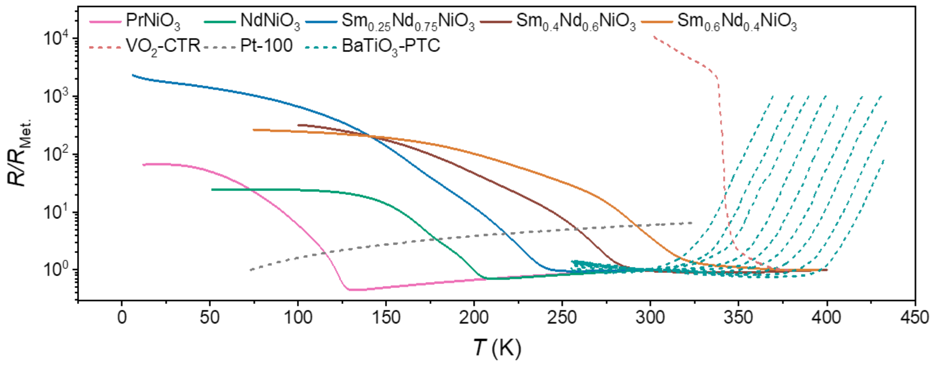

Fig. S17 | Comparing the temperature dependence in resistance for the as-made *RE*NiO_3@$LaAlO_3$ critical temperature resistance (CTR) thermistors and the reported ones based on VO_2-CTR [15], as well as the commercialized doped $BaTiO_3$ and Pt thermistors [16, 17].

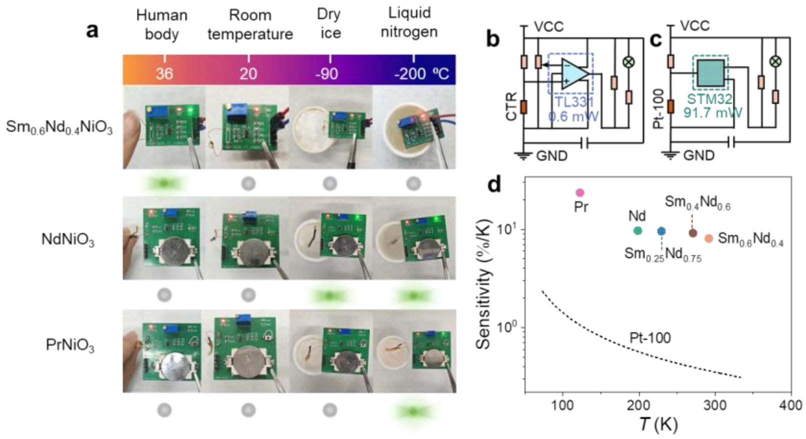

Fig. S18 | The images and superiority of as-made analog temperature alarming. (a), The three temperature alarming modules with critical temperature of body temperature, dry ice and liquid nitrogen using encapsulates of $Sm_{0.6}Nd_{0.4}NiO_3$, $NdNiO_3$, and $PrNiO_3$, respectively. These results demonstrate the temperature alarming capability of as-made modules from body temperature down to the cryogenic range. (b–d), Comparing power consumption and sensitivity with conventional cryogenic temperature sensing systems. (b), The power consumption of temperature alarming module based on the *RE*NiO_3 critical temperature resistor and comparative circuit. (c), The power consumption of temperature alarming module based on conventional Pt-thermistor connected with analog-to-digital converter. (d), Comparing the sensitivity of *RE*NiO_3-based critical temperature resistors with conventional Pt-100 thermistor in cryogenic range temperature alarming. These results demonstrate that our module exhibits two orders of magnitude lower energy consumption and one order higher sensitivity compared with analog to digital converter.

Section 4. Film growths of *RE*NiO_3 films on sapphire substrates.

In section 4, more results are provided for *RE*NiO_3 grown on sapphire substrates. Fig. S19 shows more results for calculations of interfacial energy and differential charge density via the density functional theory. Fig. S20 shows the crystal structure and electrical transportations of $NdNiO_3$ films grown on sapphire and Al_2O_3-buffered silicon. Fig. S21 shows more morphologies of $NdNiO_3/Al_2O_3$ (0001). Fig. S22 shows the metal-insulator transition behavior of *RE*NiO_3 films grown on sapphire (0001). Fig. S23 shows the determination of resistive switch of the metal-insulator transition property of *RE*NiO_3 films grown on sapphire (0001). Fig. S24 shows the resistive switch across hydrogenation induced Mottronic transition of *RE*NiO_3 grown on Al_2O_3-buffered silicon.

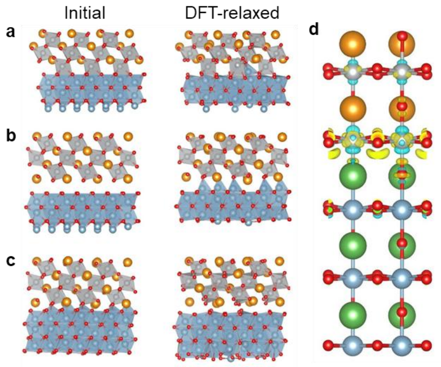

Fig. S19 | Additional results for calculations of interfacial energy and differential charge density via the density functional theory. (a–c), Three kinds of possible $NdNiO_3/Al_2O_3$ interfaces are constructed, initial and fully relaxed, performed by density functional theory (DFT). The DFT relaxations of first and second interfaces are quite difficult to converge and exhibit obvious interface reconstruction, indicating their instability. For the third interfaces, the reconstruction is quite slight, and the octahedral character of both $NdNiO_3$ and Al_2O_3 is maintained, and thus is adopted in our calculations. (d), Differential charge density of $NdNiO_3/LaAlO_3$ interface (isosurface value of 0.01 e/Bohr^3). The magnitude in charge density of $NdNiO_3/Al_2O_3$ interface is substantially larger than $NdNiO_3/LaAlO_3$. The calculation result provides a theoretical evidence for the oriented growth of nickelates films on sapphire.

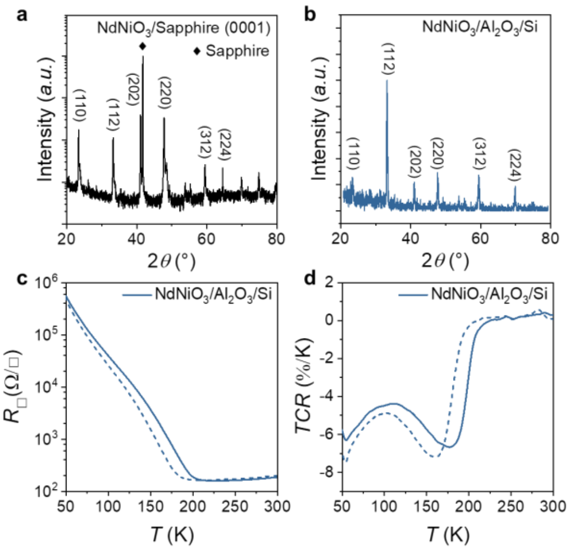

Fig. S20 | Crystal structure and electrical transportations of $NdNiO_3$ films grown on sapphire and Al_2O_3-buffered silicon. (a), The X-ray diffraction (XRD) pattern of as-grown $NdNiO_3$/sapphire (0001). (b), The grazing-incidence XRD patterns of the as-grown $NdNiO_3/Al_2O_3/Si$. (c), The sheet resistance as measured as a function of temperature for $NdNiO_3/Al_2O_3/Si$. (d), The temperature coefficient of resistance (*TCR*) plotted as a function of temperature for $NdNiO_3/Al_2O_3/Si$. These results demonstrate the compatibility in growing nickelates films on both sapphire and silicon substrates via the present molten-salt assisted liquid phase epitaxy strategy.

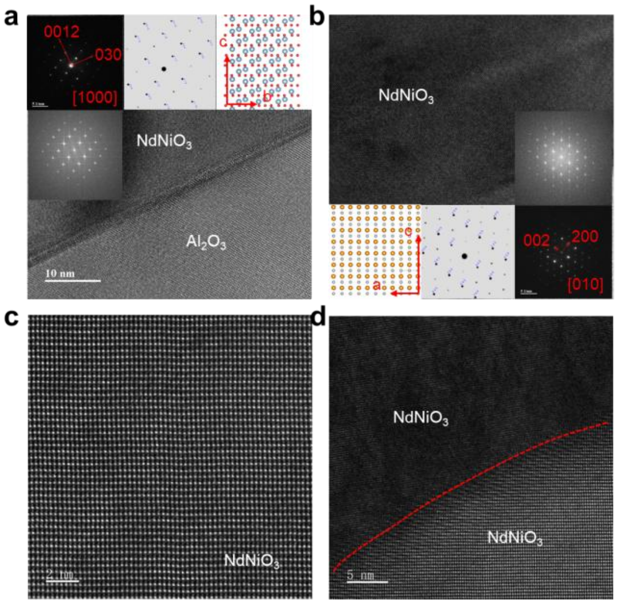

Fig. S21 | The additional morphologies of $NdNiO_3/Al_2O_3$ (0001) film. (a), The cross-section morphology of $NdNiO_3/Al_2O_3$ (0001) aligned with the crystallographic axis of Al_2O_3 and selected area electron diffraction (SAED) result of Al_2O_3. (b), The morphology of $NdNiO_3$ aligned with the crystallographic axes of $NdNiO_3$ at a low magnification and SAED result of $NdNiO_3$. (c), The morphology of $NdNiO_3$ at a high magnification. (d), The grain boundary in $NdNiO_3$, marked with red line. These results demonstrate the oriented growth of $NdNiO_3$ film on sapphire via molten-salt assisted liquid phase epitaxy strategy.

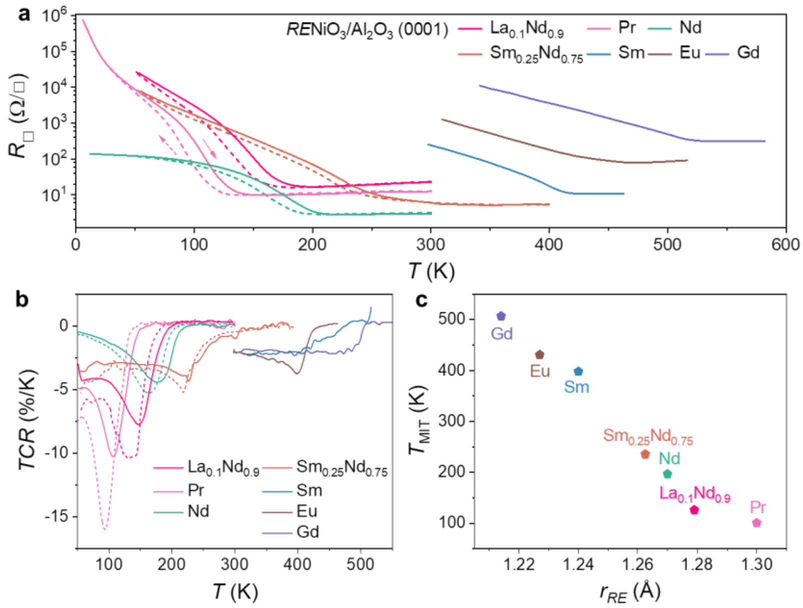

Fig. S22 | The metal-insulator transition behavior of *RE*NiO_3 films grown on sapphire (0001). (a), The temperature dependence in sheet resistance as measured for as-grown *RE*NiO_3/sapphire (0001). (b), The temperature coefficient of resistance (*TCR*) plotted as a function of temperature. (c), The critical temperature associated with metal-insulator transition (T_{MIT}) plotted as a function of the radius of rare-earth (r_{RE}). The above results demonstrate that abrupt and widely tunable metal-insulator transition functionalities are also achievable for growing nickelates on sapphire using the present molten-salt assisted liquid phase epitaxy strategy.

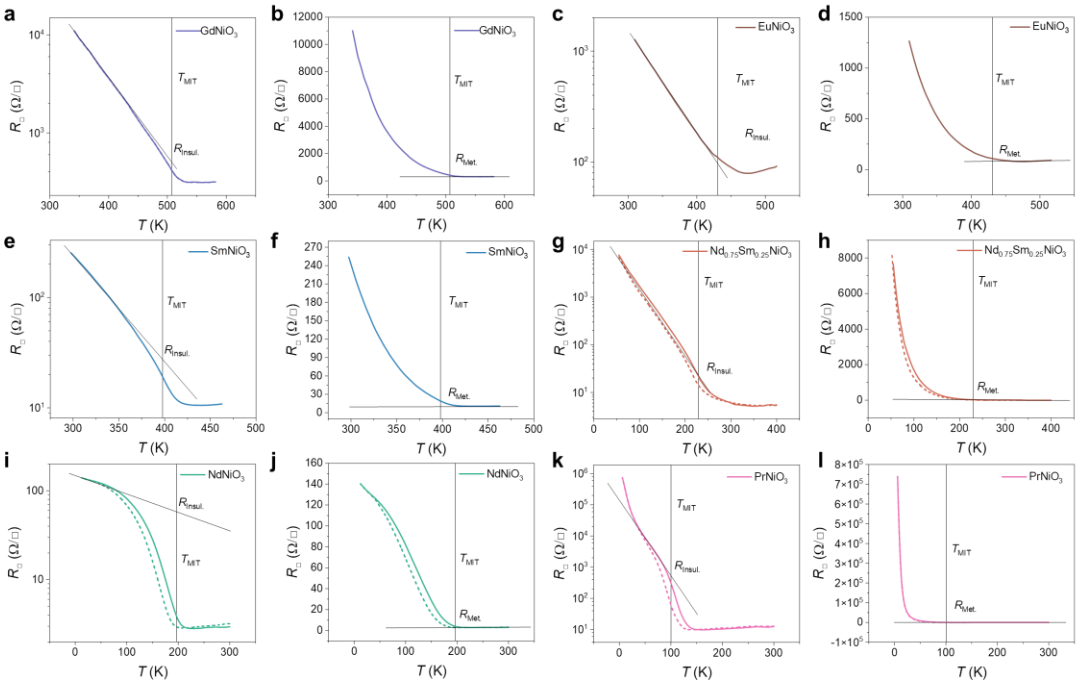

Fig. S23 | Determination of the resistive change across critical temperature ($R_{Insul.}/R_{Met.}$) of $RE$$NiO_3/Al_2O_3$ (0001) films. (a–l), The $R_{Insul.}$ and $R_{Met.}$ across critical temperature associated with metal-insulator transition (T_{MIT}) of $RE$$NiO_3/Al_2O_3$ (0001) films. (a) and (b), $GdNiO_3$. (c) and (d) $EuNiO_3$. (e) and (f) $SmNiO_3$. (g) and (h) $Nd_{0.75}Sm_{0.25}NiO_3$. (i) and (j) $NdNiO_3$. (k) and (l) $PrNiO_3$. To calculate the $R_{Insul.}$ and $R_{Met.}$, linear fitting was performed on the insulating and metallic phases on the sheet resistance-temperature ($R_{\square}$-T) tendencies. Subsequently, the sheet resistances at the intersection point of the two linear fits with T_{MIT} were considered as $R_{Insul.}$ and $R_{Met.}$, respectively. The $R_{Insul.}/R_{Met.}$ was determined as $R_{Insul.}$ divided by $R_{Met.}$.

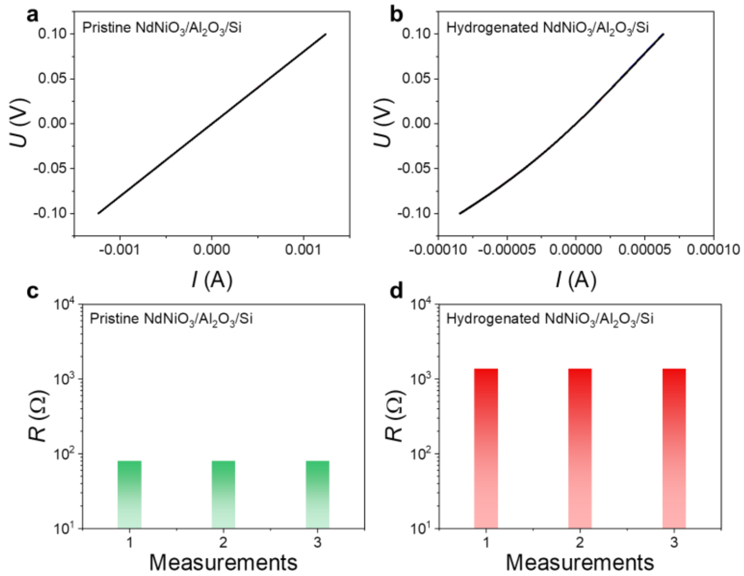

Fig. S24 | The Mottronic properties of $NdNiO_3$ grown on Al_2O_3 buffered Si. (a–b), The representative current-voltage (*I*-*V*) curve measured for (a) the pristine and (b) the hydrogenated $NdNiO_3$ with platinum patterns. (c–d), The resistances of $NdNiO_3$ with top platinum patterns (c) before and (d) after the hydrogenation process. The hydrogenation elevates the resistivity of $NdNiO_3$ by one order, demonstrating the Mottronic functionality as triggered by protonation achieved in as-grown $NdNiO_3/Al_2O_3/Si$.

Section 5. Wafer-scale film growth of *RE*NiO$_3$.

In section 5, more results are provided for the ambient growth of *RE*NiO$_3$ films on sapphire with scalability. Fig. S25 demonstrates the temperature dependence of *RE*NiO$_3$ (*RE*: Pr, Nd, Sm, Eu, Gd) grown in air or under flowing oxygen for using eutectic KCl-LiCl molten salt. Fig. S26 demonstrate the uniformity of metal-insulator transition functionality for as-grown *RE*NiO$_3$/sapphire wafers.

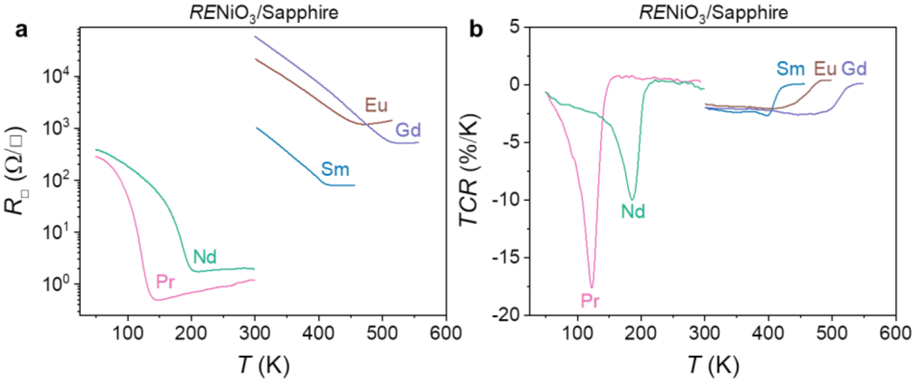

Fig. S25 | Metal-insulator transition behavior of as-grown PrNiO$_3$, NdNiO$_3$, SmNiO$_3$, EuNiO$_3$ and GdNiO$_3$ on sapphire substrate in air or under ambient pressure of flowing oxygen. (a) The sheet resistance measured as a function of temperature for *RE*NiO$_3$/sapphire. (b) The temperature coefficient of resistance (*TCR*) for *RE*NiO$_3$/sapphire. The abrupt metal-insulator transition behaviors are similar to the ones grown at MPa-high oxygen pressures.

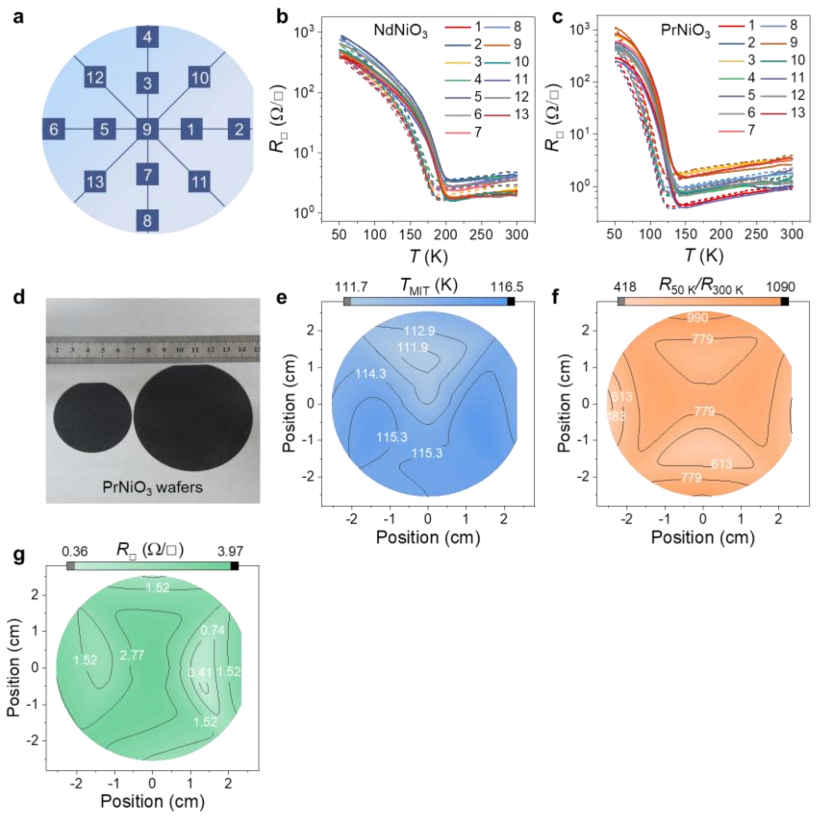

Fig. S26 | Characterizing the uniformity of metal-insulator transition functionality for as-grown *RE*NiO_3/sapphire wafers. (a), The schematic illustration indicates the regions, from where small pieces of samples were taken for further characterizations in order to evaluate the homogeneity in metal-insulator transition (MIT) functionality of the wafer. (b–c), The temperature dependent sheet resistance as measured for as-grown 2-inch (b), $NdNiO_3$ and (c), $PrNiO_3$ wafers, respectively. (d), The representative images of as-grown 2-inch and 3-inch $PrNiO_3$ wafers. (e–g), Wafer-scaled mapping of MIT properties of the as-grown 2-inch $PrNiO_3$ film: (e) the critical temperature associated with metal-insulator transition (T_{MIT}), (f) the resistive change from 50 K to 300 K ($R_{50\ K}/R_{300\ K}$), (g) the sheet resistance at room temperature. The above results demonstrate the high uniformity of MIT for as-grown *RE*NiO_3 wafers.

Section 6. Extending the alkali-metal halide molten-salts assisted liquid phase epitaxy strategy to the µm-thick film growth of other functional oxides.

In section 6, we further extended the alkali-metal halide molten-salts assisted liquid phase epitaxy strategy for growing more material systems. Fig. S27 shows the X-ray diffraction patterns and functionality of $Pr_{0.67}Sr_{0.33}MnO_3$, $SrRuO_3$, and $Bi_2Sr_2CaCu_2O_8$.

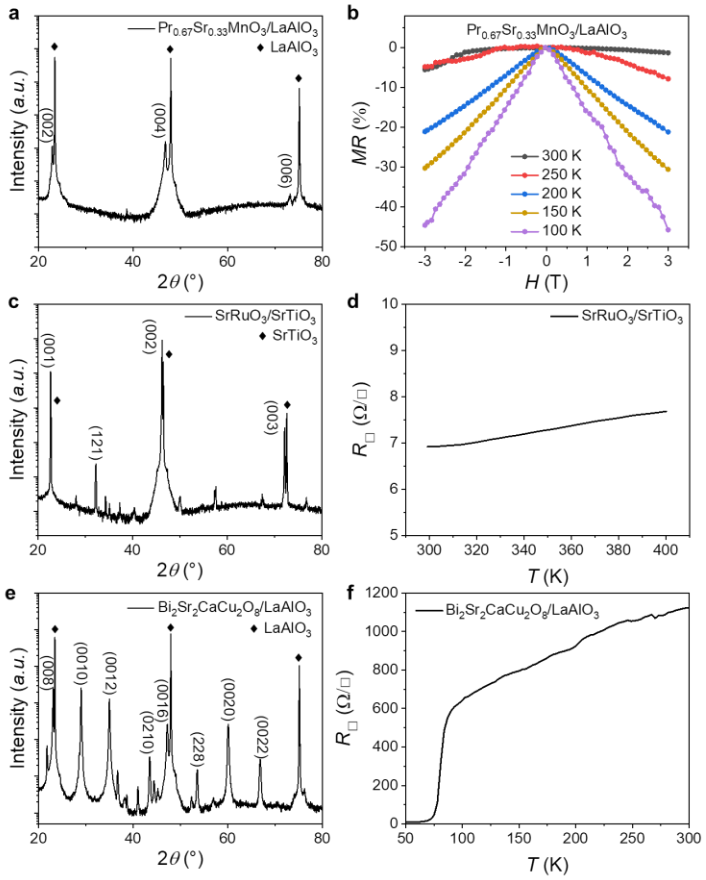

Fig. S27 | Extending the molten-salt assisted liquid phase epitaxy strategy to growing other multicomponent oxides covering a wealthy of functionalities. (a), The X-ray diffraction (XRD) pattern for as-grown $Pr_{0.67}Sr_{0.33}MnO_3/LaAlO_3$ (001) using KCl-molten salt assisted liquid phase epitaxy strategy in air at 800°C. (b), The magnetoresistance (MR) plotted as a function of magnetic field for $Pr_{0.67}Sr_{0.33}MnO_3/LaAlO_3$ (001) as measured in different temperatures. (c), The XRD patterns of as-grown $SrRuO_3/SrTiO_3$ (001) using KCl-molten salt assisted liquid phase epitaxy strategy in air at 800°C. (d), The sheet resistance of $SrRuO_3$/ $SrTiO_3$ (001) plotted as a function of temperature. (e), The XRD patterns of the as-grown $Bi_2Sr_2CaCu_2O_8/LaAlO_3$ (001) using KCl-molten salt assisted liquid phase epitaxy strategy in air at 850°C. (f), The sheet resistance of $Bi_2Sr_2CaCu_2O_8/LaAlO_3$ (001) plotted as a function of temperature. The above results demonstrate that the molten-salt assisted liquid phase epitaxy strategy can be successfully extended to growing both perovskites and non-perovskites correlated systems at µm-thickness, covering a wealth of functionalities, such as superconductivity, magnetoresistance, and oxide electrode.

Section 7. More details about the growths of *RE*NiO_3 via the alkali-metal halide molten-salts assisted liquid phase epitaxy strategy.

Growth of *RE*NiO_3 under high oxygen pressures: We firstly performed the alkali-metal halide molten-salts assisted liquid phase epitaxy of *RE*NiO_3 using KCl molten salt under MPa oxygen pressure at 800°C, which contains the following five steps. Step 1) The oxides precursors (e.g., RE_2O_3, and NiO) and the molten salt of KCl were dried at 800°C for 12 h to remove the adsorbed water. Step 2) The pretreated precursors and molten salt were weighed according to the stoichiometric molar ratio (e.g., RE_2O_3: NiO: KCl= 1: 2: 1.5). Afterwards the mixture was ground in an agate mortar for 15 min. Step 3) Half of the mixed powders were transferred to the bottom of an alumina tube. Afterwards the substrate was placed on top of the powders, covered by the remaining powders, which were tightly pressed. Step 4) The alumina tube was placed in a high-pressure furnace for sintering at 800°C under 7–10 MPa oxygen pressure (e.g., 7 MPa for growing $PrNiO_3$ and $NdNiO_3$, 10 MPa for growing $SmNiO_3$, $EuNiO_3$, and $GdNiO_3$) for 24 h. Step 5) The *RE*NiO_3 films were rinsed in water by ultrasound with deionized water and alcohol at three times to remove KCl.

Growth of *RE*NiO_3 under ambient pressures: To further relieve the reliance on MPa-high oxygen pressure, we used eutectic KCl-LiCl as molten salt with descendent melting point, hence the reaction temperature can be further reduced down to 600–700°C. The entire process for ambient growth of *RE*NiO_3 contains the following five steps. Step 1) The oxides precursors (e.g., RE_2O_3, and NiO) and the molten salts of KCl and LiCl were dried at 800°C for 12 h to remove the adsorbed water. Step 2) The pretreated precursors and molten salt were weighed according to the stoichiometric molar ratio (e.g., RE_2O_3: NiO: KCl: LiCl= 2: 4: 3: 4.5). Afterwards the mixture was ground in an agate mortar for 15 min. Step 3) Half of the mixed powders were transferred to the bottom of an alumina crucible. Afterwards the substrate was placed on top of the powders, covered by the remaining powders and pressed tightly. Step 4) The alumina crucible was placed in a muffle furnace for sintering at 500–700°C for 12 h in air to grow $PrNiO_3$ and $NdNiO_3$, and under flowing oxygen to grow $SmNiO_3$, $EuNiO_3$, and $GdNiO_3$. Step 5) The *RE*NiO_3 films were rinsed in water by ultrasound with deionized water and alcohol at three times to remove the KCl-LiCl molten salts.

References

1. Jaramillo R, Schoofs F and Ha SD *et al.* High pressure synthesis of $SmNiO_3$ thin films and implications for thermodynamics of the nickelates. *Journal of Materials Chemistry C* 2013; **1**: 2455-62.
2. Alonso JA, Martínez-Lope MJ and Casais MT *et al.* Room-temperature monoclinic distortion due to charge disproportionation in $RNiO_3$ perovskites with small rare-earth cations (R=Ho, Y, Er, Tm, Yb, and Lu): A neutron diffraction study. *Physical Review B* 2000; **61**: 1756-63.
3. Escote MT, da Silva AML and Matos JR *et al.* General Properties of Polycrystalline $LnNiO_3$ (Ln=Pr, Nd, Sm) Compounds Prepared through Different Precursors. *Journal of Solid State Chemistry* 2000; **151**: 298-307.
4. Shannon RD. Revised effective ionic radii and systematic studies of interatomic distances in halides and chalcogenides. *Acta Crystallographica Section A* 1976; **32**: 751-67.
5. Chen J, Mao W and Ge B *et al.* Revealing the role of lattice distortions in the hydrogen-induced metal-insulator transition of $SmNiO_3$. *Nature Communications* 2019; **10**: 694.
6. Ha SD, Aydogdu GH and Ramanathan S. Examination of insulator regime conduction mechanisms in epitaxial and polycrystalline $SmNiO_3$ thin films. *Journal of Applied Physics* 2011; **110**: 094102.
7. K DD, Singh A and Sathapathy S *et al.* Large Magnetoresistance in $PrNiO_3$ Thin Film Deposited by RF Magnetron Sputtering. *Journal of Superconductivity and Novel Magnetism* 2023; **36**: 623-9.
8. Harisankar S, Soni K and Yadav E *et al.* Strain-mediated effects of oxygen deficiency and variation in non-Fermi liquid behavior of epitaxial $PrNiO_{3-\delta}$ thin films. *Journal of Physics: Condensed Matter* 2019; **31**: 135601.
9. Feigl L, Schultz BD and Ohya S *et al.* Structural and transport properties of epitaxial $PrNiO_3$ thin films grown by molecular beam epitaxy. *Journal of Crystal Growth* 2013; **366**: 51-4.
10. Tu L, Wen Z and Cui Y *et al.* The synthesis and resistivity of $Pr_{1-x}Sr_xNiO_{3(2)}$ films deposited on $SrTiO_3$ by the PA-CSD method. *Physica B: Condensed Matter* 2022; **636**: 413860.
11. Novojilov MA, Gorbenko OY and Graboy IE *et al.* Perovskite rare-earth nickelates in the thin-film epitaxial state. *Applied Physics Letters* 2000; **76**: 2041-2043.
12. Wang L, Stoerzinger KA and Chang L *et al.* Tuning Bifunctional Oxygen Electrocatalysts by Changing the A-Site Rare-Earth Element in Perovskite Nickelates. *Advanced Functional Materials* 2018; **28**: 1803712.
13. Hauser AJ, Mikheev E and Moreno NE *et al.* Correlation between stoichiometry, strain, and metal-insulator transitions of $NdNiO_3$ films. *Applied Physics Letters* 2015; **106**: 092014.
14. Guo Q, Farokhipoor S and Magén C *et al.* Tunable resistivity exponents in the metallic phase of epitaxial nickelates. *Nature Communications* 2020; **11**: 2949.
15. Hisao F. 半導体の応用（その 3）熱電材料. *Journal of the Japan Society of Precision Engineering* 1980; **46**: 704-709.
16. Murata Manufacturing Co L. *Murata's Posistor Addresses Overheating in Various FETs.*
17. Lake Shore Cryotronics I. PT-100 Series Platinum RTDs.
